# SFXN3 Serves as a Predictive Biomarker for Cisplatin Response and Survival in Head and Neck Squamous Cell Carcinoma

**DOI:** 10.32604/or.2026.078376

**Published:** 2026-05-21

**Authors:** Eun-Jeong Jeong, Yeon Soo Kim, Yujin Lee, Jae-Sung Ryu, Yung Hyun Choi, Eunjeong Kim

**Affiliations:** 1 Department of Otorhinolaryngology–Head and Neck Surgery, College of Medicine, Konyang University, Daejeon, Republic of Korea; 2 Department of Otorhinolaryngology–Head and Neck Surgery, College of Medicine, Korea University, Seoul, Republic of Korea; 3 BK21 FOUR KNU Creative BioResearch Group, Department of Biology, College of Natural Sciences, Kyungpook National University, Daegu, Republic of Korea; 4 New Drug Development Center, Osong Medical Innovation Foundation, Cheongju, Republic of Korea; 5 Basic Research Laboratory for the Regulation of Microplastic-Mediated Diseases and Anti-Aging Research Center, Dong-eui University, Busan, Republic of Korea; 6 Department of Biochemistry, College of Korean Medicine, Dong-eui University, Busan, Republic of Korea; 7 Center for Genome Engineering, Institute for Basic Science, Daejeon, Republic of Korea

**Keywords:** Sideroflexin 3 (SFXN3), head and neck squamous cell carcinoma (HNSCC), bioinformatics, The Cancer Genome Atlas (TCGA), biomarker, prognosis, tumor microenvironment

## Abstract

**Objective**: The systematic evaluation of expansive genomic databases facilitates the discovery of clinically vital biomarkers. While Sideroflexin 3 (SFXN3) consistently displays elevated expression in head and neck squamous cell carcinoma (HNSCC), its specific pathobiological functions and prognostic value remain insufficiently characterized. This study aims to delineate the clinical and functional significance of SFXN3 in HNSCC. **Methods**: We interrogated SFXN3 expression patterns, patient survival outcomes, and immune cell infiltration characteristics utilizing multiple independent repositories, including the cancer genome atlas (TCGA) and gene expression omnibus (GEO). The prognostic independence of SFXN3 was verified via multivariate Cox regression. These computational findings were subsequently corroborated through targeted *in vitro* functional assays. **Results**: SFXN3 expression was significantly augmented in HNSCC, demonstrating strong correlations with advanced disease stages and reduced overall survival. Multivariate models confirmed its status as an independent prognostic indicator. Furthermore, SFXN3 upregulation was closely tied to an immunosuppressive microenvironment. *In vitro* validations revealed that SFXN3 knockdown substantially impairs cell proliferation while simultaneously sensitizing HNSCC cells to cisplatin-induced apoptosis via intrinsic pathways. **Conclusion**: Ultimately, SFXN3 represents a robust prognostic indicator and an actionable therapeutic vulnerability to counteract both drug resistance and tumor immune escape in HNSCC.

## 1 Introduction

Head and neck squamous cell carcinoma (HNSCC) remains a remarkably heterogeneous and aggressive malignancy. Despite continuous therapeutic innovations encompassing targeted therapies, immunotherapies, and surgical refinements, patient survival rates remain suboptimal due to rapid progression and frequent metastasis [1,2]. Consequently, the identification of dependable molecular targets to precisely predict patient outcomes and tailor therapeutic strategies is of paramount clinical importance [3]. Specifically, the development of chemoresistance, particularly to cisplatin, and the presence of a highly immunosuppressive tumor microenvironment are major hurdles in HNSCC management. Understanding the underlying molecular networks driving these processes is critical for developing novel targeted strategies.

Recent literature strongly implicates metabolic reprogramming as a foundational driver of HNSCC [4]. Alterations in mitochondrial dynamics and metabolism not only fuel the high energetic demands of rapidly proliferating tumor cells but also critically modulate apoptotic pathways and immune cell infiltration [5]. Consequently, mitochondrial membrane proteins, which regulate the flux of metabolites essential for cellular homeostasis, are increasingly recognized as potential drivers of oncogenesis and therapeutic resistance [6].

Sideroflexin 3 (SFXN3) is a mitochondrial inner-membrane protein of the evolutionarily conserved sideroflexin (SFXN) family, a group of multi-pass transporters predominantly expressed in metabolically active tissues. SFXN family members are involved in amino-acid–dependent mitochondrial metabolism and cellular stress-response pathways [7,8]. For example, SFXN1 is a recognized serine transporter critical for redox homeostasis and one-carbon metabolism [9]. In contrast, the biological role of SFXN3 remains poorly understood. Current evidence indicates that SFXN3 is highly expressed in neurons and contributes to mitochondrial organization, neuronal development, and the regulation of protein networks associated with neurodegeneration and cell-death pathways [10,11]. However, its potential roles in cancer biology have been explored only recently, with studies suggesting that SFXN family dysregulation may be associated with tumor progression in specific malignancies [8,12].

Considering the critical influence of mitochondrial function on tumor proliferation, chemoresistance, and immune modulation, we postulated that SFXN3 acts as an unrecognized driver of HNSCC pathogenesis. Therefore, this study aims to perform a comprehensive pan-cancer screening combined with multi-database clinical validation to assess the prognostic and biological significance of SFXN3, and to explore its functional role and associations with the immune microenvironment in HNSCC.

## 2 Materials and Methods

### 2.1 GEPIA3 (Gene Expression Profiling Interactive Analysis 3)

The GEPIA3 web portal (https://gepia3.bioinfoliu.com/) was utilized to evaluate the clinical implications of SFXN3. This platform successfully amalgamates RNA-seq datasets from both The Genotype-Tissue Expression (GTEx) and the Cancer Genome Atlas (TCGA) projects. For our primary differential expression evaluation, we aggregated 520 HNSCC tumor samples and 44 normal controls. The analysis workflow involved: (i) contrasting SFXN3 transcript levels between normal and malignant cohorts; (ii) constructing Kaplan-Meier survival curves to assess overall survival (OS); and (iii) calculating Spearman’s correlation coefficients between SFXN3 and EGFR. Following the standard GEPIA3 pipeline, raw expression data were normalized using a log_2_ (TPM + 1) transformation. Strict statistical thresholds were maintained, setting the absolute log_2_ fold-change (|log_2_ FC|) cutoff at 1.0 and the significance level at *p* < 0.01.

### 2.2 Microarray Data

Transcriptomic profiles were extracted from the publicly accessible Gene Expression Omnibus (GEO) repository (http://www.ncbi.nlm.nih.gov/geo/). GEO is a publicly available database that archives a wide array of gene expression profiles generated across different experimental platforms and biological conditions. Employing a systematic search methodology as previously documented [13], we queried the database through 28 October 2024. The search strategy employed the keywords “head and neck squamous cell carcinoma” [Title/Abstract] and “Homo sapiens” [Organism]. The search included datasets published up to 28 October 2024. To ensure robust differential expression analysis, we applied the following strict inclusion criteria: (1) studies must contain genome-wide expression profiling data (e.g., microarray or RNA-seq); (2) datasets must include paired or comparative samples of primary HNSCC tumor tissues and adjacent normal tissues; and (3) adequate sample sizes were required to ensure statistical power. Datasets lacking appropriate normal controls, non-human studies, or those based exclusively on cell lines without clinical samples were filtered out. Consequently, three datasets—GSE58911 [14], GSE178537 [15,16], and GSE33232 [17]—were deemed suitable and incorporated into our downstream analyses (Table 1). These cohorts supplied comprehensive transcriptomic data, enabling the evaluation of *SFXN3* transcript levels and their correlation with the clinicopathological characteristics of HNSCC.

**Table 1:** Summary of human HNSCC transcriptomic datasets retrieved from the Gene Expression Omnibus (GEO).

GEO	Platform	Sample	Normal	Tumor	Metastasis	Reference
GSE 58911	GPL6244	Head and neck squamous cell carcinoma, homo sapiens	15	15		Lobert et al. [14]
GSE 178537	GPL16791 GPL18573	Head and neck squamous cell carcinoma, homo sapiens	20	19	9	Cheng et al. [15,16]
GSE 33232	GPL5175 GPL6801 GPL8490 GPL8786	Head and neck squamous cell carcinoma, homo sapiens	25	44		Stansfield et al. [17]

GEO, gene expression omnibus; HNSCC, head and neck squamous cell carcinoma.

### 2.3 Kaplan–Meier Plotter Analysis

SFXN3 prognostic significance was assessed using the Kaplan–Meier Plotter platform (http://kmplot.com/analysis/, accessed 03 December 2025). This comprehensive resource curates survival metrics from over 35,000 tumor samples spanning 21 malignancies. This resource was employed to analyze the correlation between *SFXN3* transcript abundance and the prognostic trajectories of HNSCC patients. Survival trajectories were plotted utilizing the Kaplan-Meier methodology, while group disparities (high vs. low expression) were statistically assessed via the log-rank test. The magnitude of the prognostic effect was quantified by calculating hazard ratios (HRs) alongside their 95% confidence intervals (CIs) for OS. Because our evaluation was specifically targeted at a single pre-selected gene (*SFXN3*), multiple testing corrections for the generated log-rank *p*-values were deemed unnecessary. For survival assessments stratified by immune cell presence, the relative abundance of tumor-infiltrating immune cells was estimated using the platform’s integrated xCell algorithm [18,19]. Patients were categorized based on their enrichment scores: the “immune cell-enriched” group comprised the upper quartile (top 25%), while the “immune cell-decreased” group consisted of the lower quartile (bottom 25%). Specifically, due to the available data modules in the platform, the macrophage-related analyses represent the total macrophage population, without further sub-stratification into M1 or M2 phenotypes.

### 2.4 UALCAN

UALCAN database (http://ualcan.path.uab.edu/, accessed 03 December 2025) was employed to systematically interrogate TCGA-derived multi-omics data. UALCAN, an open-access tool integrating TCGA datasets, enables comprehensive exploration of gene expression across multiple cancer types and clinically relevant patient subgroups. Here, we utilized UALCAN to investigate SFXN3 expression patterns in HNSCC and its association with clinical factors. Clinical subgroups were defined based on the pathological tumor stages (Stages 1–4) and histological tumor grades (Grades 1–4) provided by the TCGA clinical data annotations. To determine the statistical significance of mRNA expression variances between normal controls and specific tumor subgroups, we applied Student’s *t*-test with Welch’s correction, consistent with UALCAN’s standardized analytical framework. In addition to transcriptomic profiling, proteomic analysis of SFXN3 was performed using UALCAN, which integrates mass spectrometry–based datasets generated via the International Cancer Proteogenomic Consortium and Clinical Proteomic Tumor Analysis Consortium (CPTAC). These resources provide harmonized protein abundance profiles for multiple cancers, including HNSCC, generated using standardized TMT-labeling and deep proteome quantification pipelines. The CPTAC HNSCC dataset was queried via UALCAN to evaluate SFXN3 protein expression, comparing primary tumor tissues (n = 108) with normal control tissues (n = 71). Cases with missing proteomic quantification values were excluded. In alignment with the UALCAN CPTAC pipeline, protein expression levels were quantified as Z-values, which are derived from internally and cross-sample normalized log_2_ spectral count ratios. Significant differences in protein expression between the specified groups were evaluated via an unpaired two-sample *t*-test with statistical significance defined as *p* < 0.05. This proteomic analysis was conducted to corroborate RNA-level observations and confirm that SFXN3 dysregulation in HNSCC extends to the protein level.

### 2.5 Transfection for Small Interfering RNA

Prior to the targeted silencing of SFXN3, FaDu cells were seeded into 24-well (2 × 10^4^ cells/well) or 6-well (1 × 10^5^ cells/well) culture dishes and grown to approximately 80% confluency. We procured a specific small interfering RNA (siRNA) against SFXN3, together with a non-targeting negative control (NC) siRNA, which was supplied by Bioneer (Daejeon, Republic of Korea). For gene silencing, the designated siRNAs (30 nM) were introduced into the cells using the Lipofectamine RNAiMAX reagent (Thermo Fisher Scientific, Waltham, MA, USA; Cat. No.13778), following the standard guidelines supplied by the manufacturer. Briefly, siRNA-lipid complexes were pre-incubated before being added to the culture medium. Transfected cells were maintained for 48 h, at which point SFXN3 knockdown was confirmed by qRT-PCR and Western Blotting. Subsequently, these cells were harvested for further functional analyses and molecular evaluations. The specific sequences of the siRNAs are provided in Table 2.

**Table 2:** Detailed oligonucleotide sequences of the siRNAs employed in the current investigation.

Gene	Sense (5′-3′)	Antisense (5′-3′)
*siNC*	UUCUCCGAACGUGUCACGU	ACGUGACACGUUCGGAGAA
*si*SFXN3	GAGACUUAUAGACACUAGU	ACUAGUGUCUAUAAGUCUC

siRNA, small interfering RNA.

### 2.6 RNA Isolation and Quantitative Real-Time Polymerase Chain Reaction (qRT-PCR)

Total cellular RNA was isolated utilizing the Nucleozol reagent (Macherey-Nagel GmbH & Co. KG, Düren, Germany; Cat. No. 740404.200) according to the manufacturer’s instructions. RNA yield and purity were subsequently verified via a NanoDrop One UV-Vis spectrophotometer (Thermo Fisher Scientific, Grand Island, NY, USA; Cat. No. ND-ONE); only samples with an A_260_/A_280_ ratio of 1.9–2.1 and an A_260_/A_230_ ratio of 2.0–2.2 were used for further analysis. We synthesized complementary DNA (cDNA) from 500 ng of high-quality total RNA employing the PrimeScript™ RT Reagent Kit (Takara, Kusatsu, Shiga, Japan; Cat. No. RR037A) according to the protocol of the manufacturer. Quantitative Real-Time PCR (qRT-PCR) was conducted on a StepOnePlus Real-Time PCR system (Applied Biosystems, Waltham, MA, USA) using SYBR Green Master Mix (Applied Biosystems; Cat. No. 4309155). The amplification parameters consisted of an initial denaturation phase at 95°C for 10 min, followed sequentially by 40 amplification cycles (95°C for 15 s, and 60°C for 30 s). Target gene expression was normalized against the endogenous reference gene, β-actin. The oligonucleotide primer sequences utilized are detailed in Table 3.

**Table 3:** Summary of qRT-PCR primers utilized in this study.

Gene	Sense (5′-3′)	Antisense (5′-3′)
SFXN3	AGCTAGAGGAGGAGGCCAA	TCCTGGAGAGATTGAATCAGCC
TOMM22	CAGTCCCCGGACGAATTGC	CGACAGGGTCTCATCTAGCTC
ACAT1	GGAGCCAGGATTGTTGGTCA	AGAAGCACCTCCTCCTCCAT
SLC25A38	GTCCCCTTCCATTGTGAGATG	CCTCGCAAGAAATACTGCTTCA
β-actin	TCCTCTCCCAAGTCCACACAGG	GGGCACGAAGGCTCATCATTC

Sideroflexin 3 (SFXN3); Translocase of Outer Mitochondrial Membrane 22 (TOMM22); Acetyl-CoA Acetyltransferase 1 (ACAT1); Solute Carrier Family 25 Member 38 (SLC25A38); Quantitative real-time polymerase chain reaction (qRT-PCR), qRT-PCR.

### 2.7 Cell Culturing

Human HNSCC cell lines, including FaDu and Detroit562, were obtained from the American Type Culture Collection (ATCC, Manassas, VA, USA; Cat. No. HTB-43 and CCL-138) and propagated in Minimum Essential Medium (Welgene, Gyeongsan, Republic of Korea; Cat. No. LM007-01) supplemented with 10% fetal bovine serum (FBS, Thermo Fisher Scientific, Grand Island, NY, USA; Cat. No. 16000), 1% penicillin–streptomycin (Thermo Fisher Scientific, Grand Island, NY, USA; Cat. No. 10378016), and 1% sodium pyruvate (Thermo Fisher Scientific, Grand Island, NY, USA; Cat. No. 11360070). SCC9 and SCC25 cells (ATCC, Manassas, VA, USA; Cat. No. CRL-1628) were cultured in DMEM/F12 medium (Thermo Fisher Scientific, Grand Island, NY, USA; Cat. No. 11320033) supplemented with 10% FBS, 1% penicillin–streptomycin, and 1% sodium pyruvate. The YD9 cell line was obtained from the Korean Cell Line Bank (Seoul, Republic of Korea; Cat. No. 60549) and maintained in Roswell Park Memorial Institute-1640 (Welgene, Gyeongsan, Republic of Korea; Cat. No. LM011-01) fortified with 10% FBS, 1% penicillin–streptomycin, and 1% sodium pyruvate. Authentication was guaranteed by the provider (ATCC) via STR profiling, and cells were used within 6 months of resuscitation. Furthermore, the cultures were routinely monitored for mycoplasma contamination utilizing an e-Myco™ Mycoplasma PCR Detection Kit (ver. 2.0, INTRON, Seongnam, Republic of Korea; Cat. No. 25235) to definitively confirm the absence of infection throughout the experimental timeframe. Cultures were maintained at 37°C in a humidified atmosphere with 5% CO_2_.

### 2.8 Cell Proliferation Assay

To determine the basal cellular proliferation rates of the SFXN3-depleted FaDu and SCC25 models, the EZ-Cytox Cell Viability Assay Kit (Dogenbio, Seoul, Republic of Korea; Cat. No. EZ-3000) was utilized. Cells were seeded into 96-well culture plates at a density of 3 × 10^3^ cells/well in 100 μL of complete culture medium and incubated at 37°C with 5% CO_2_. Cellular proliferation was evaluated at 24, 48, and 72 h post-seeding. At each designated time point, 10 μL of EZ-Cytox reagent was added to each well, followed by an additional incubation for 1–2 h according to the manufacturer’s recommendations. The absorbance of the formazan product was measured at 450 nm using a microplate spectrophotometer (Synergy HTX, BioTek, Winooski, VT, USA).

### 2.9 Cell Viability Assay

To assess cell viability and sensitivity following cisplatin treatment, cells were seeded into 96-well plates at a density of 3 × 10^3^ cells/well and allowed to adhere overnight. The following day, the cells were treated with cisplatin (Sigma-Aldrich, St. Louis, MO, USA; Cat. No. 15663-27-1) at the indicated concentrations from 0 to 10 μM. Following a 72-h incubation period with the drug, 10 μL of EZ-Cytox reagent was added to each well. After a 1–2 h incubation, the absorbance was measured at 450 nm using a microplate spectrophotometer. Cell viability was calculated as a percentage relative to the absorbance of the untreated control cells.

### 2.10 Evaluation of Caspase 3/7 Activity

Apoptotic induction was evaluated by measuring Caspase-3/7 activity. FaDu and SCC25 cells, previously transfected with siSFXN3 or control siRNA, were plated at 3 × 10^3^ cells/well in opaque white 96-well plates. Following overnight adherence, the cells were challenged with cisplatin for 72 h. Caspase activity was subsequently quantified utilizing the Caspase-Glo^®^ 3/7 Assay Kit (Promega, Mannheim, Germany; Cat. No. G8092) according to the manufacturer’s protocol. An equivalent volume of the luminescent assay reagent was dispensed into each well. After a 1-h ambient temperature incubation, luminescent intensity was measured on a microplate spectrophotometer (Synergy HTX, BioTek Instruments, Winooski, VT, USA).

### 2.11 Colony Formation Analysis

The clonogenic survival capacity of SFXN3-depleted cells was determined via colony formation assays. Both FaDu and SCC25 cells were plated at a low density of 300 cells per well utilizing 6-well culture plates (SPL Life Sciences, Pocheon, Republic of Korea; Cat. No. 30006). After allowing 48 h for initial growth, the cultures were exposed to 1 μM cisplatin. The cells were then maintained in culture for an additional 14 days to allow for macroscopic colony development. Post-incubation, the resulting colonies were fixed using methanol, stained with a 0.1% crystal violet solution (Sigma-Aldrich, St. Louis, MO, USA; Cat. No. V5265). Visible colonies were counted to evaluate the clonogenic potential of SFXN3-deficient cells.

### 2.12 Flow Cytometry Analysis

Apoptotic cell fractions were quantified using flow cytometry. Twenty-four hours post-transfection with siSFXN3, and cisplatin was administered 24 h after transfection. After 72 h of cisplatin treatment, all cells were harvested, washed with cold PBS, and stained utilizing an Annexin V–FITC/PI dual-labeling kit (BD Biosciences, San Jose, CA, USA; Cat. No. 556547) for 15 min in the dark, strictly following the manufacturer’s instructions. Each run included unstained, single-stained, and compensation controls. Flow cytometric measurements were performed using a FACS system (Beckman Coulter, Brea, CA, USA). Forward scatter (FSC) and side scatter (SSC) parameters were used to exclude debris, and doublets were eliminated using FSC-area versus FSC-height gating. For every individual sample, at least 10,000 cellular events were acquired. Apoptotic cell populations were determined based on Annexin V–FITC and propidium iodide (PI) staining patterns. Cells negative for both Annexin V and PI (Annexin V−/PI−) were considered viable, Annexin V-positive and PI-negative cells (Annexin V+/PI−) were classified as early apoptotic, and Annexin V-positive and PI-positive cells (Annexin V+/PI+) were defined as late apoptotic or necrotic. Gating thresholds were established using unstained and single-stained controls. Data analysis was executed using CytExpert software (version 2.6; Beckman Coulter, Inc., Brea, CA, USA), and standardized gating procedures were applied to quantify apoptotic populations.

### 2.13 Western Blotting

To isolate total cellular proteins, HNSCC cells were disrupted in RIPA lysis buffer (Thermo Fisher Scientific, Grand Island, NY, USA; Cat. No. 89901) enriched with EDTA (Thermo Fisher Scientific, Grand Island, NY, USA; Cat. No. R1021), protease inhibitors (Thermo Fisher Scientific, Grand Island, NY, USA; Cat. No. 78440), and phosphatase inhibitors (Thermo Fisher Scientific, Grand Island, NY, USA; Cat. No. 78440). The cell lysates were incubated on ice for 30 min and subsequently clarified via centrifugation (15,000× *g*, 30 min). Total protein concentrations were determined employing a BCA assay kit (Thermo Fisher Scientific, Grand Island, NY, USA; Cat. No. 23227). Protein aliquots (20 μg) were denatured, resolved on 4–12% gradient SDS–PAGE using a Mini Gel Tank system (Thermo Fisher Scientific, Grand Island, NY, USA; Cat. No. A25977) and subsequently transferred onto PVDF membranes via the iBlot dry blottin, according to the manufacturer’s instructions. To prevent non-specific binding, the membranes were blocked with 5% bovine serum albumin (BSA, Bovogen Biologicals, Keilor East, VIC, Australia; Cat. No. BSAS 0.1) for 1 h at ambient temperature. Following overnight incubation at 4°C with specific primary antibodies, the blots were washed with Tris-buffered saline containing 0.1% Tween-20, membranes were probed with HRP-conjugated secondary antibodies for 2 h at room temperature. Immunoreactive protein bands were visualized employing SuperSignal West Femto Maximum Sensitivity Substrate (Thermo Fisher Scientific, Grand Island, NY, USA; 34095), according to the manufacturer’s instructions. Detailed information regarding the antibodies employed in this study is provided in Table 4.

**Table 4:** List of antibodies used in this study.

Antibody	Company/City, Province/State, Country	Catalogue No.	Dilution Ratios
SFXN3	Abnova Biotechnology, Taipei, Taiwan	H00081855-K	1:1000
PARP	Cell Signaling Technology, Danvers, MA, USA	9542	1:1000
Cleaved Caspase 3	Cell Signaling Technology, Danvers, MA, USA	9661	1:1000
Cleaved Caspase 9	Cell Signaling Technology, Danvers, MA, USA	9501	1:1000
GAPDH	Cell Signaling Technology, Danvers, MA, USA	5174	1:1000
Anti-rabbit IgG	Cell Signaling Technology, Danvers, MA, USA	7074	1:2000
Anti-mouse IgG,	Cell Signaling Technology, Danvers, MA, USA	7076	1:2000

### 2.14 Protein-Protein Interaction (PPI) Network Analysis

To map the functional protein interaction landscape of SFXN3, we utilized the STRING biological database (version 12.0; https://string-db.org/). Employing “SFXN3” as the primary query within the Homo sapiens genome context, the network was constructed by integrating diverse interaction sources, including experimental evidence, co-expression profiles, text mining, and curated databases. To ensure the reliability of the predicted interactions, the minimum confidence threshold was configured to a medium score (>0.400).

### 2.15 Statistical Analysis

All statistical evaluations were conducted utilizing GraphPad Prism software (version 5.0; GraphPad Software, San Diego, CA, USA). For multiple group comparisons, a one-way analysis of variance (ANOVA) followed by Tukey’s post hoc test was performed. Where appropriate, pairwise comparisons were analyzed using Student’s *t*-tests. For the flow cytometric apoptosis data, a two-way ANOVA was implemented to evaluate the main effects and interactions of the experimental treatments, followed by Tukey’s and Šídák’s multiple comparisons tests. The independent prognostic significance of clinical variables was ascertained via multivariate Cox proportional hazards regression models. Quantitative data are consistently presented as the mean ± standard deviation (SD). The threshold for statistical significance was established at *p* < 0.05.

## 3 Results

### 3.1 Characterization and Validation of Sideroflexin 3 Expression across Diverse Cancer Types

**Figure 1:**
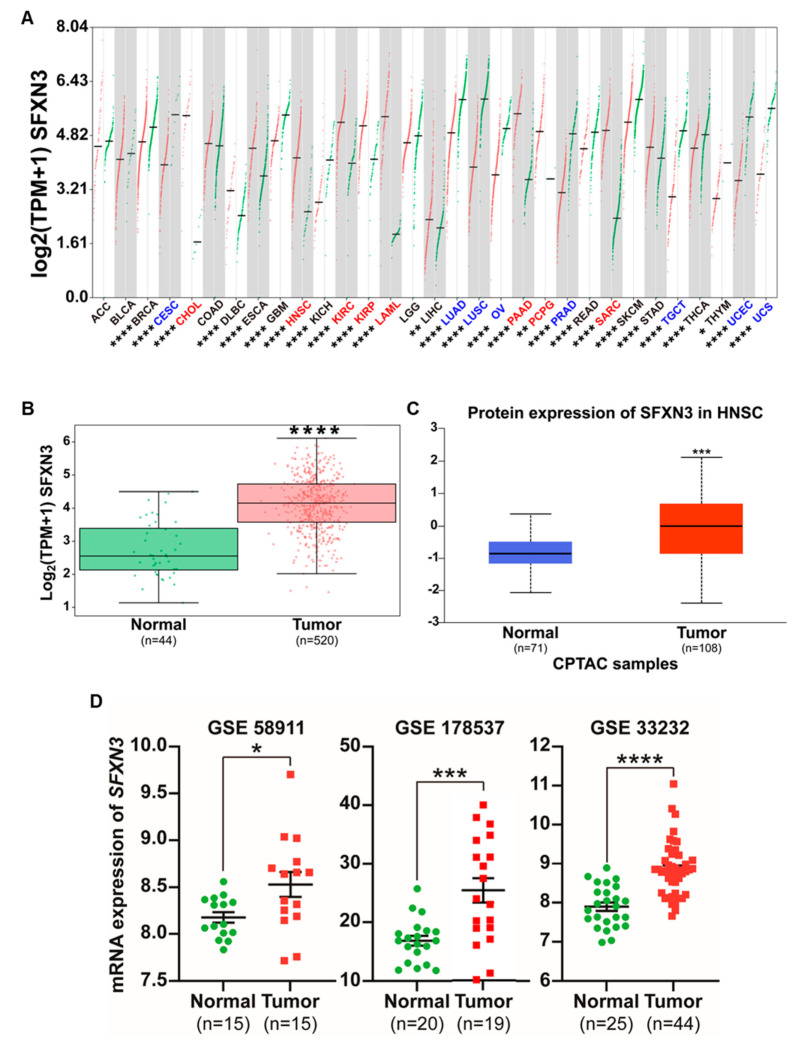
Sideroflexin 3 (SFXN3) is significantly upregulated in Head and Neck Squamous Cell Carcinoma (HNSCC) and across multiple cancer types. (**A**) Pan-cancer analysis of SFXN3 mRNA expression across TCGA and GTEx datasets using the GEPIA3 platform. Expression levels (log2 [TPM + 1]) are shown for paired tumor (red) and normal (green) tissues across various cancer types, revealing widespread upregulation in tumors. (**B**) Differential SFXN3 expression in HNSCC based on GEPIA3 analysis. Tumor samples (n = 520) show significantly higher SFXN3 mRNA levels compared to normal tissues (n = 44). (**C**) SFXN3 protein expression in HNSCC was analyzed using CPTAC proteomics data accessed through UALCAN. Tumor tissues (n = 108) show significantly increased SFXN3 protein abundance compared to normal controls (n = 71). (**D**) Validation of SFXN3 overexpression in independent GEO cohorts. Datasets GSE58911 (normal n = 15; tumor n = 15), GSE178537 (normal n = 20; tumor n = 19), and GSE33232 (normal n = 25; tumor n = 44), indicating significantly higher SFXN3 transcript levels in tumor specimens. Statistical significance is indicated as *p < 0.05, **p < 0.01, ***p < 0.001, ****p < 0.0001.

Using the GEPIA3 platform, we systematically analyzed SFXN3 expression across diverse malignancies and observed pronounced upregulation in tumor tissues compared with normal tissues in multiple cancer types, including Cholangiocarcinoma (CHOL), Kidney renal clear cell carcinoma (KIRC), Kidney renal papillary cell carcinoma (KIRP), Acute myeloid leukemia (LAML), Pancreatic adenocarcinoma (PAAD), Pheochromocytoma and Paraganglioma (PCPG), Sarcoma (SARC), and HNSCC (Fig. 1A). SFXN3 expression was significantly higher in HNSCC tumor specimens (n = 520) than in normal tissue (n = 44; Fig. 1B, *p* < 0.05).

To extend these transcriptomic findings to the protein level, SFXN3 expression was evaluated using CPTAC proteomic data. Consistent with mRNA-based analyses, SFXN3 protein abundance was significantly higher in HNSCC tumor samples compared to normal tissues (Fig. 1C). We independently validated these findings by analyzing multiple GEO datasets. Consistent with the TCGA-based results, several GEO cohorts (GSE58911, GSE178537, and GSE33232) showed significantly higher SFXN3 mRNA expression in tumor tissues compared to normal tissues (Fig. 1D). Collectively, consistent SFXN3 upregulation at transcriptomic and proteomic levels across independent datasets and analytical platforms highlights its robustness dysregulation in HNSCC and supports its potential biological relevance in tumor-associated processes.

### 3.2 Association of Sideroflexin 3 Expression with Tumor Stage and Histological Grade in Head and Neck Squamous Cell Carcinoma

To further assess the clinical relevance of SFXN3 expression in HNSCC, we utilized the UALCAN platform to examine its relationship with key pathological parameters, including tumor stage and histological grade.

**Figure 2:**
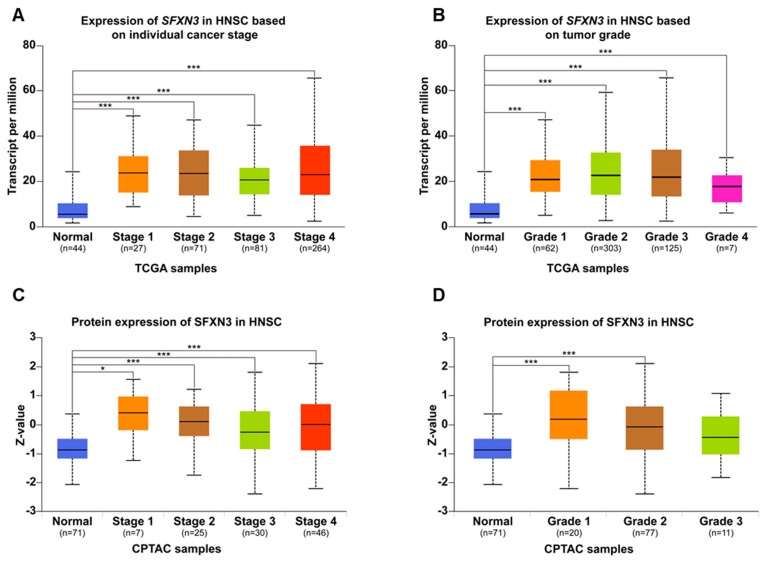
SFXN3 expression in HNSCC across pathological stages and tumor grades. (**A**) TCGA analysis of *SFXN3* mRNA expression across HNSCC stages. TPM was significantly elevated in tumor tissues from stages 1–4 compared to normal tissues (normal n = 44; stage 1 n = 27; stage 2 n = 71; stage 3 n = 81; stage 4 n = 264). (**B**) TCGA-based *SFXN3* mRNA expression stratified based on tumor grade. *SFXN3* transcript abundance remained consistently higher in grades 1–3 compared to normal tissues (normal n = 44; grade 1 n = 62; grade 2 n = 303; grade 3 n = 125; grade 4 n = 7). (**C**) CPTAC proteomic analysis of SFXN3 expression across HNSCC stages. Protein levels (Z-values) were significantly increased in tumor samples compared to normal tissues (normal n = 71; stage 1 n = 7; stage 2 n = 25; stage 3 n = 30; stage 4 n = 46), with stage 1 tumors exhibiting the highest protein abundance. (**D**) CPTAC proteomic analysis of SFXN3 expression across tumor grades. Protein expression was elevated in grades 1–3 compared to normal tissues (normal n = 71; grade 1 n = 20; grade 2 n = 77; grade 3 n = 11), with grade 1 exhibiting the strongest upregulation. Statistical significance is indicated as *p < 0.05 ***p < 0.001.

Transcriptomic profiling derived from the TCGA cohort indicated that *SFXN3* transcripts are profoundly upregulated in HNSCC tissues relative to normal counterparts, regardless of the clinical stage (I–IV) or tumor grade (1–4) (Fig. 2A,B). SFXN3 transcriptional upregulation was consistent across stages and grades, with no stepwise increase observed with disease progression.

Consistent with the transcriptomic data, CPTAC-based proteomic analyses revealed that the abundance of SFXN3 protein was markedly elevated in neoplastic tissues compared to the normal control group (Fig. 2C,D). However, protein abundance did not follow a linear trend with tumor stage or grade. The highest SFXN3 levels were observed in stage I and grade 1 tumors, with slightly lower but similar expression in more advanced stages and higher grades.

Collectively, these findings indicate that SFXN3 upregulation occurs early during HNSCC development and persists across different pathological stages and grades, rather than increasing progressively with disease severity. This stage- and grade-independent expression pattern suggests that SFXN3 dysregulation is an early molecular event in HNSCC and warrants further investigation into its biological and clinical implications.

### 3.3 Association between SFXN3 Transcript Levels and Patient Survival in Head and Neck Squamous Cell Carcinoma

**Figure 3:**
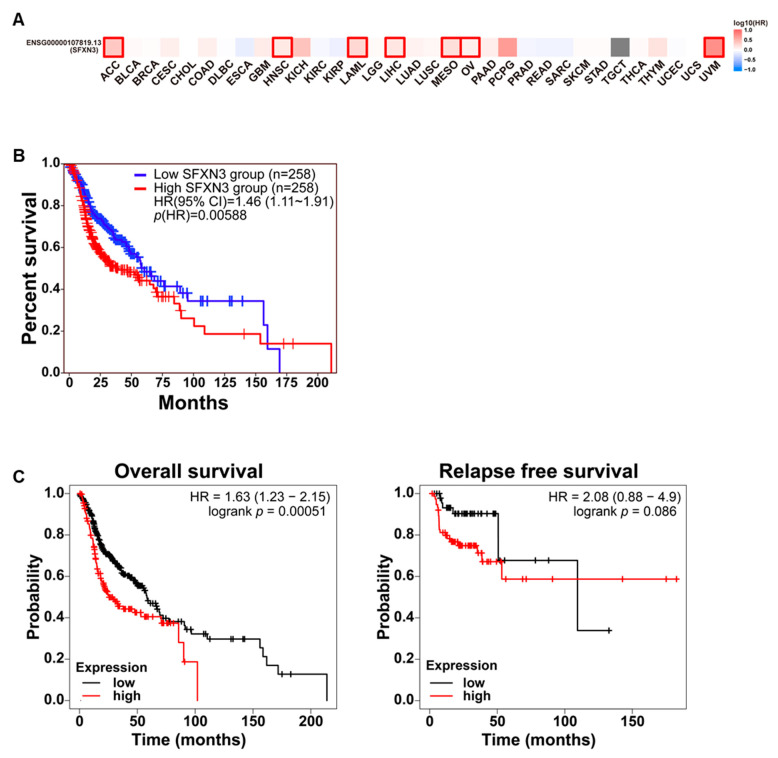
High SFXN3 expression is associated with poor survival outcomes in HNSCC. (**A**) GEPIA3 pan-cancer survival map depicting the prognostic significance of SFXN3 across multiple tumor types. High SFXN3 expression correlates with reduced survival probabilities in HNSCC and LIHC, indicated by red-boxed. (**B**) Kaplan–Meier Overall survival (OS) analysis of patients with HNSCC from TCGA, stratified into high- and low-expression groups (n = 129 per group). Patients with elevated SFXN3 expression had significantly worse OS (Hazard Ratio (HR) = 1.91, 95% Confidence Interval (CI): 1.27–2.87; *p* = 0.00179). (**C**) Independent validation using Kaplan–Meier Plotter datasets. High SFXN3 expression was associated with reduced OS (HR = 1.63, 95% CI: 1.23–2.15; log-rank *p* = 0.00051) and showed a trend toward poorer relapse-free survival (HR = 2.08, 95% CI: 0.88–4.9; log-rank *p* = 0.086).

To determine the clinical significance of SFXN3 expression in HNSCC, Kaplan-Meier survival assessments were conducted across several distinct patient cohorts. GEPIA3-based survival mapping showed that high SFXN3 expression was linked to reduced OS in patients with HNSCC (Fig. 3A). Similarly, GEPIA-based OS analysis revealed significantly poorer outcomes in patients with high SFXN3 expression (HR = 1.46, 95% CI: 1.11–1.97, *p* = 0.00588) (Fig. 3B).

These findings were further validated using independent Kaplan–Meier survival analyses. Elevated SFXN3 levels significantly correlated with decreased OS (HR = 1.63, 95% CI: 1.23–2.15, log-rank *p* = 0.00051), while relapse-free survival exhibited a statistically nonsignificant tendency towards inferior outcomes (HR = 2.08, 95% CI: 0.88–4.90, log-rank *p* = 0.086) (Fig. 3C).

**Figure 4:**
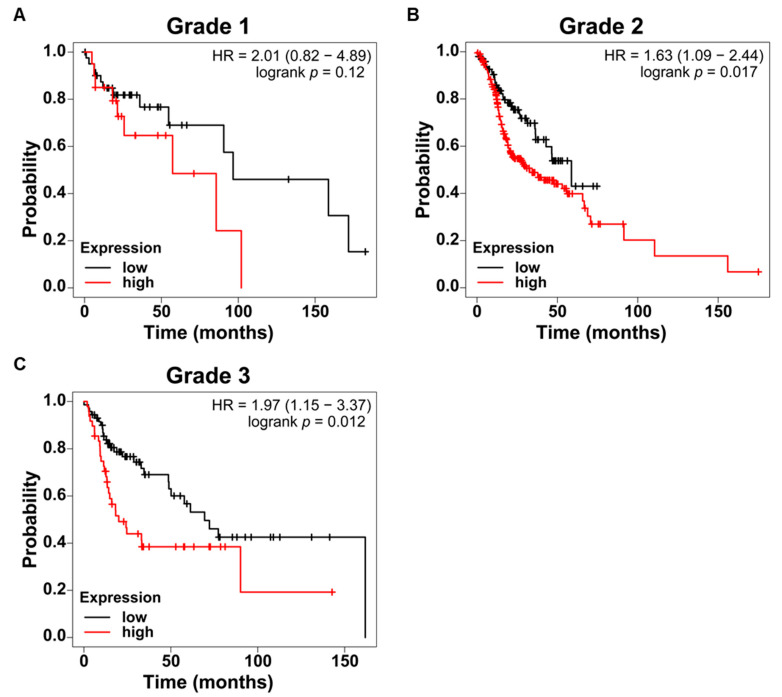
Prognostic effect of SFXN3 expression across tumor grades in HNSCC. (**A**) Kaplan–Meier OS analysis of grade I patients with HNSCC stratified based on SFXN3 expression. High SFXN3 expression showed a trend toward poorer survival, but the difference was not statistically significant (HR = 2.01, 95% CI: 0.82–4.89; log-rank *p* = 0.12). (**B**) In grade II tumors, elevated SFXN3 expression was significantly associated with reduced OS (HR = 1.63, 95% CI: 1.09–2.44; *p* = 0.017), indicating a stronger prognostic effect with increasing tumor aggressiveness. (**C**) Grade III tumors showed the most pronounced survival separation, with the SFXN3-high group exhibiting significantly poorer outcomes (HR = 1.97, 95% CI: 1.15–3.37; *p* = 0.012).

Grade-stratified survival analyses revealed that the prognostic effect of SFXN3 expression was stronger in higher-grade tumors. Grade I tumors exhibited a nonsignificant trend toward reduced survival in the high-expression group (HR = 2.01, 95% CI: 0.82–4.89, *p* = 0.12) (Fig. 4A), while significant survival differences were observed in grade II (HR = 1.63, 95% CI: 1.09–2.44, *p* = 0.017) and grade III tumors (HR = 1.97, 95% CI: 1.15–3.37, *p* = 0.012) (Fig. 4B,C).

Sex-stratified analyses revealed that high SFXN3 expression was associated with poorer OS in male (HR = 1.63, 95% CI: 1.17–2.27, *p* = 0.0035) and female patients (HR = 1.83, 95% CI: 1.13–2.97, *p* = 0.013) (Fig. S1A,B). Stage-stratified survival analyses revealed no significant differences in stages I–III, but high SFXN3 expression was linked to a pronounced and statistically significant survival disadvantage in stage IV disease (HR = 1.80, 95% CI: 1.26–2.57, *p* = 0.00096) (Fig. S1C–F). Collectively, these findings indicate that high SFXN3 expression is consistently associated with poorer survival outcomes in HNSCC, particularly in higher-grade and advanced-stage tumors.

To determine whether SFXN3 is an independent prognostic factor, we performed a multivariate Cox regression analysis adjusting for age, gender, race, tumor stage, and tumor purity. The analysis confirmed that high SFXN3 expression remains independently associated with poorer overall survival (HR = 1.239, 95% CI: 1.023–1.500, *p* = 0.028) (Supplementary Table S1).

### 3.4 Association between Sideroflexin 3 Expression and Immune Cell–Enriched Tumor Contexts in Head and Neck Squamous Cell Carcinoma

**Figure 5:**
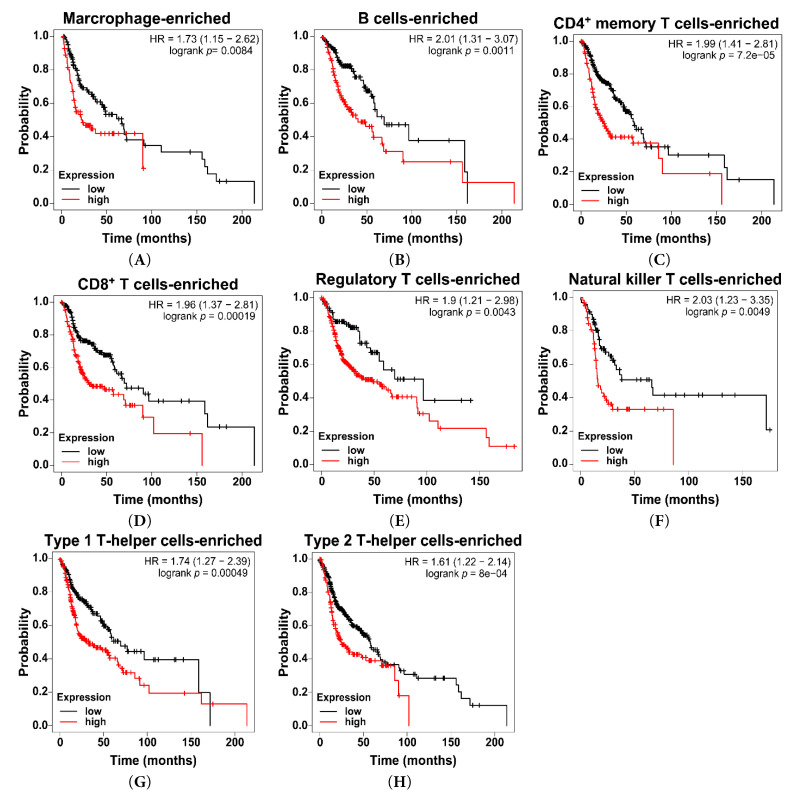
High SFXN3 expression predicts poor OS across immune cell–enriched HNSCC subgroups. (**A**) Total macrophage-enriched tumors: high SFXN3 expression was associated with significantly worse OS (HR = 1.73; *p* = 0.0084). (**B**) B-cell–enriched tumors: high SFXN3 predicted markedly reduced OS (HR = 2.01; *p* = 0.0011). (**C**) Cluster of differentiation 4-positive (CD4^+^) memory T-cell–enriched tumors: high SFXN3 associated with poor OS (HR = 1.99; *p* = 0.000072). (**D**) Cluster of differentiation 8-positive (CD8^+^) memory T-cell–enriched tumors: high SFXN3 associated with decreased OS (HR = 1.96; *p* = 0.00019). (**E**) Regulatory T-cell (Treg)–enriched tumors: high SFXN3 predicted shorter OS (HR = 1.90; *p* = 0.0043). (**F**) Natural killer T cell–enriched tumors: high SFXN3 associated with poorer prognosis (HR = 2.03; *p* = 0.0049). (**G**) Type 1 T helper cells-enriched tumors: high SFXN3 correlated with reduced OS (HR = 1.74; *p* = 0.00049). (**H**) Type 2 T helper cells-enriched tumors: high SFXN3 correlated with inferior OS (HR = 1.61; *p* = 0.0008).

To further explore how SFXN3 expression relates to the tumor immune microenvironment, we evaluated its prognostic significance in immune cell–enriched tumor subsets using Kaplan–Meier survival analysis. Given the key role of tumor-infiltrating immune cells in influencing clinical outcomes, survival analyses were stratified based on the enrichment of specific immune cell populations. Across all immune cell–enriched subgroups, high SFXN3 expression was consistently linked to poorer OS (Fig. 5). In total macrophage-enriched tumors, elevated SFXN3 levels were strongly linked to a substantially increased likelihood of mortality (HR = 1.73, *p* = 0.0084) (Fig. 5A). A stronger survival disadvantage was observed in B-cell–enriched tumors (HR = 2.01, *p* = 0.0011) (Fig. 5B). High SFXN3 expression was also linked to reduced survival in CD4^+^ memory T cells (HR = 1.99, *p* = 7.2 × 10^−^^5^) and CD8^+^ T cell-enriched tumors (HR = 1.96, *p* = 0.00019) (Fig. 5C,D). This adverse prognostic effect was also observed in regulatory T cell–enriched (HR = 1.90, *p* = 0.0043) and natural killer T cell–enriched tumors (HR = 2.03, *p* = 0.0049) (Fig. 5E,F). Furthermore, tumors enriched for type 1 and type 2 T helper cells similarly showed significantly poorer survival in patients with high SFXN3 expression (Th1: HR = 1.74, *p* = 0.00049; Th2: HR = 1.61, *p* = 8 × 10^−^^4^) (Fig. 5G,H). Elevated SFXN3 expression negatively impacts survival outcomes not only in immune cell–enriched subgroups but also some immune cell–decreased subgroups (Fig. S2).

Collectively, these findings indicate that the adverse prognostic effect of high SFXN3 expression persists across diverse innate and adaptive immune cell–enriched tumor contexts in HNSCC.

### 3.5 Association between Sideroflexin 3 Expression and Epidermal Growth Factor Receptor Levels in Head and Neck Squamous Cell Carcinoma

**Figure 6:**
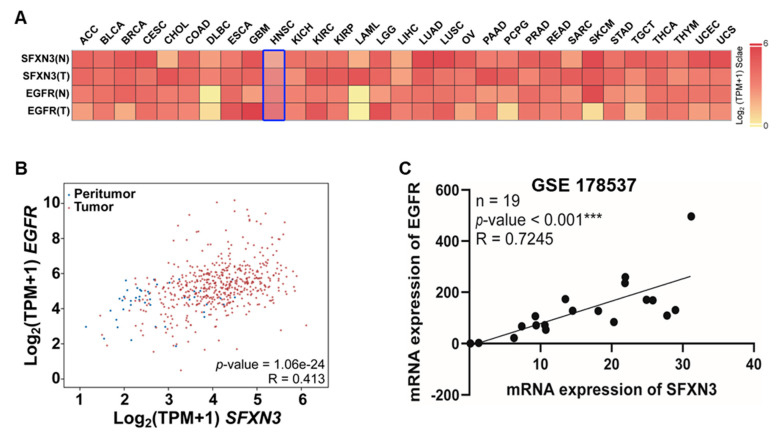
Association of SFXN3 levels with Epidermal Growth Factor Receptor (EGFR) expression in HNSCC. (**A**) Expression levels of SFXN3 and EGFR across multiple cancer types, including DLBC, ESCA, KIRC, KIRP, PAAD, SARC, STAD, and HNSCC. Both genes were markedly upregulated in tumor tissues compared to normal controls, with pronounced overexpression in HNSCC. (**B**) Scatter plot illustrating a significant positive correlation between SFXN3 and EGFR expression in HNSCC based on TCGA data. (**C**) Validation of the SFXN3–EGFR correlation in the GEO dataset GSE178537, indicating consistent and statistically significant positive associations. Statistical significance is indicated as ***p < 0.001.

To examine the link between SFXN3 expression and epidermal growth factor receptor (EGFR) signaling in HNSCC, we analyzed their correlation across multiple datasets. Evaluation of the pan-cancer heatmap demonstrated that SFXN3 is consistently upregulated in malignant tissues relative to normal controls across various cancer types, notably DLBC, ESCA, KIRC, KIRP, PAAD, SARC, STAD, and HNSCC (Fig. 6A). SFXN3 and EGFR expression levels were higher in HNSCC tumor samples than in normal tissues.

Correlation analysis of TCGA HNSCC samples showed a significant positive association between SFXN3 and EGFR expression (R = 0.413, *p* = 1.06 × 10^−24^; Fig. 6B). This relationship was independently confirmed in the GSE178537 cohort, further demonstrating a robust positive association between the mRNA levels of SFXN3 and EGFR (R = 0.7245, *p* < 0.001; n = 19; Fig. 6C).

Collectively, these findings indicate that SFXN3 expression is consistently elevated alongside EGFR expression in HNSCC across independent transcriptomic datasets, suggesting coordinated gene expression within tumor tissues.

### 3.6 Sideroflexin 3 Depletion Suppresses HNSCC Cell Growth and Enhances Cisplatin-Induced Apoptosis

**Figure 7:**
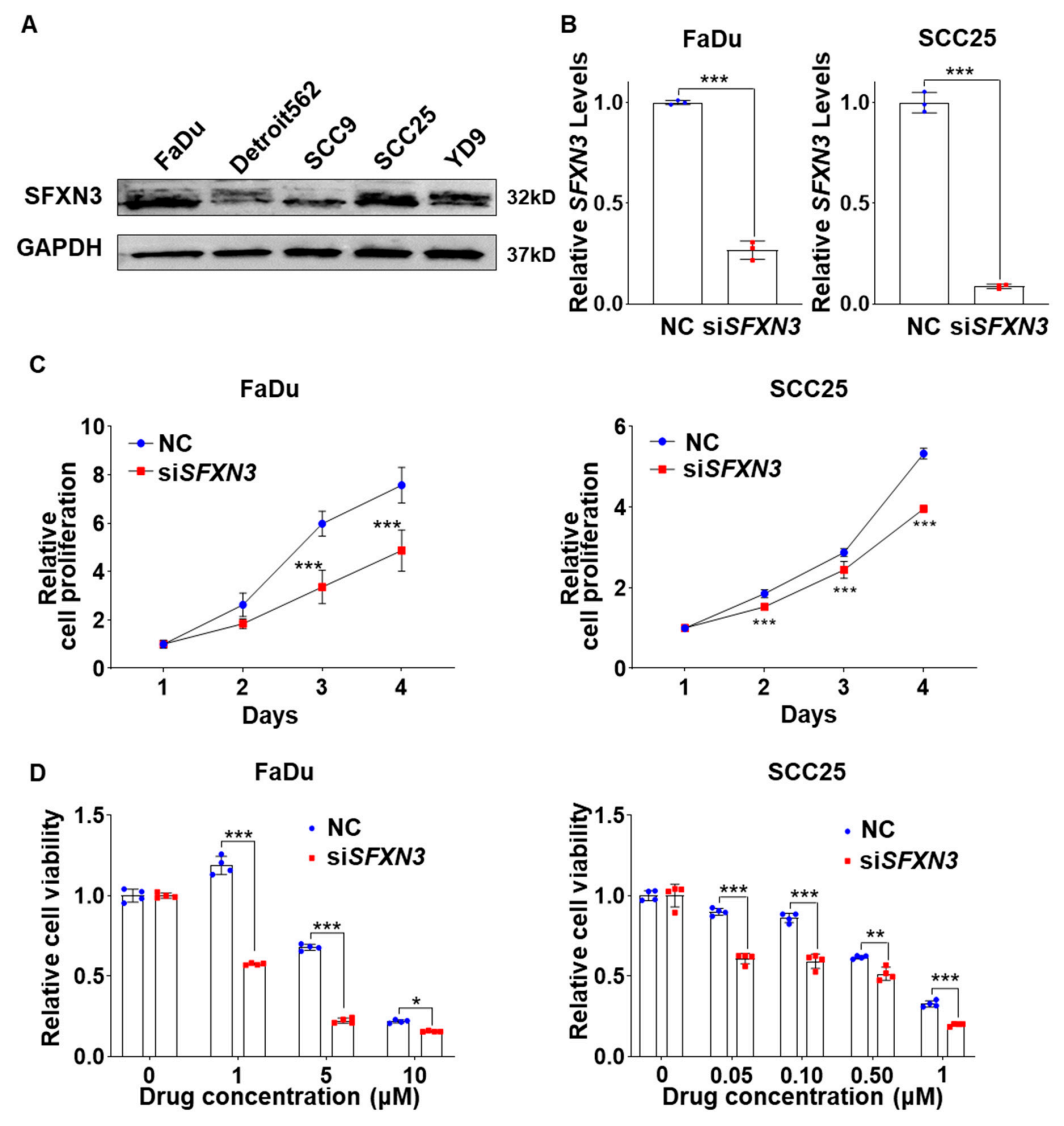
SFXN3 knockdowns suppress proliferation and enhance cisplatin sensitivity in HNSCC. (**A**) Basal protein expression levels of SFXN3 in a panel of head and neck squamous cell carcinoma (HNSCC) cell lines were examined by Western blot analysis. GAPDH was used as a loading control. (**B**) Efficient knockdown of SFXN3 in FaDu and SCC25 cells following transfection with siSFXN3 was confirmed by Western blotting and quantified relative to negative control siRNA (NC), n = 3. (**C**) Targeted knockdown of SFXN3 resulted in a significant, time-dependent suppression of proliferation in FaDu and SCC25 cells relative to control groups. (**D**) Cell viability assays demonstrated that depletion of SFXN3 markedly increased sensitivity to cisplatin treatment in FaDu and SCC25 cells across increasing drug concentrations, n = 4. Data are presented as mean ± Standard error of the mean (SEM) from three independent biological replicates. Statistical analyses were performed using two-way ANOVA with Tukey’s multiple comparisons test; detailed statistical results are provided in the Supplementary Tables S2 and S3. Asterisks denote the levels of statistical significance: *p < 0.05, **p < 0.01, and ***p < 0.001.

To elucidated the role of SFXN3 in HNSCC, we first evaluated SFXN3 protein expression across a panel of HNSCC cell line. Western blot analysis demonstrated that SFXN3 was abundantly expressed in HNSCC cells, including FaDu and SCC25 (Fig. 7A). Targeted silencing of SFXN3 using siRNA resulted in a marked reduction of SFXN3 protein levels in both cell lines compared with negative control siRNA (NC)-treated cells, confirming efficient knockdown (Fig. 7B). Functional assays revealed that suppression of SFXN3 significantly attenuated cell proliferation in both FaDu and SCC25 cells in a time-dependent manner (Fig. 7C). In addition, depletion of SFXN3 markedly increased cellular sensitivity to cisplatin, as evidenced by reduced cell viability across increasing drug concentrations (Fig. 7D).

Consistent with these findings, long-term clonogenic survival assays further demonstrated that SFXN3 knockdown significantly impaired colony-forming ability in FaDu and SCC25 cells. Notably, the inhibitory effect of SFXN3 depletion on colony formation was further enhanced in the presence of cisplatin treatment, whereas SFXN3 knockdown alone also resulted in a moderate but significant reduction in colony-forming capacity compared with control cells (Fig. S3).

**Figure 8:**
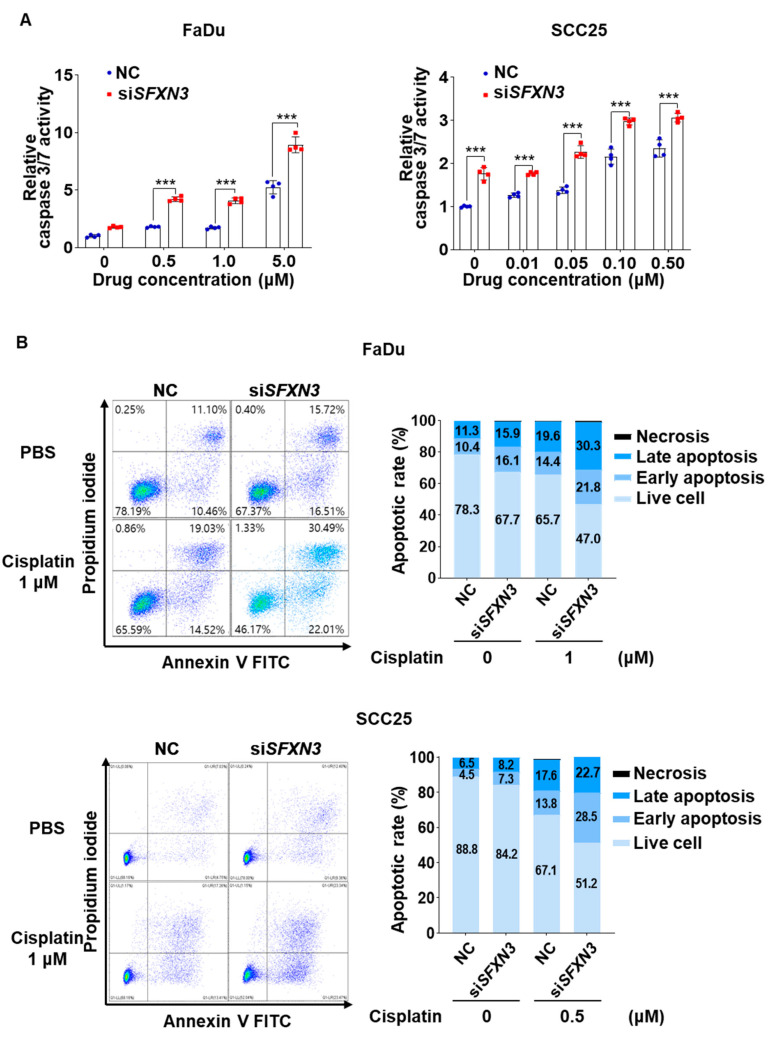
SFXN3 depletion enhances cisplatin-induced apoptosis in HNSCC. (**A**) Caspase-3/7 activity was measured in FaDu (left) and SCC25 (right) cells transfected with negative control siRNA (NC) or si*SFXN3* following treatment with increasing concentrations of cisplatin. *SFXN3* knockdown significantly enhanced cisplatin-induced caspase-3/7 activation in a dose-dependent manner. (**B**) Flow cytometric evaluation of apoptosis using Annexin V–FITC and PI staining in FaDu and SCC25 cells, which were transfected with either NC or siSFXN3 prior to cisplatin exposure (FaDu, 1 μM; SCC25, 0.5 μM). Representative flow cytometry plots (left) and quantitative analyses of live, early apoptotic, late apoptotic, and necrotic cell populations (right) are shown. Data are presented as mean ± standard deviation (SD). Statistical significance was determined by two-way ANOVA followed by Šídák’s multiple comparisons test. Statistical significance is indicated as ***p < 0.001.

Given the observed effects of SFXN3 knockdown on cell growth and clonogenic survival, we next investigated whether SFXN3 depletion influenced apoptotic cell death. Caspase-3/7 activity assays demonstrated that cisplatin induced a dose-dependent increase in caspase-3/7 activity in both FaDu and SCC25 cells, which was significantly augmented by SFXN3 knockdown (Fig. 8A).

Flow cytometric analysis using Annexin V–FITC and propidium iodide (PI) staining further confirmed that the knockdown of SFXN3 led to a substantial rise in the proportions of both early and late apoptotic cells within FaDu and SCC25 lines upon cisplatin exposure, accompanied by a reduction in the viable cell fraction (Fig. 8B).

Collectively, these results indicate that SFXN3 supports HNSCC cell proliferation and clonogenic survival, and that its suppression enhances cisplatin-induced apoptotic cell death, at least in part through increased activation of caspase-3/7–dependent apoptotic pathways.

### 3.7 Cell Line Specific Association of SFXN3 with Mitochondrial-Related Gene Expression and Cisplatin-Induced Apoptosis in HNSCC

**Figure 9:**
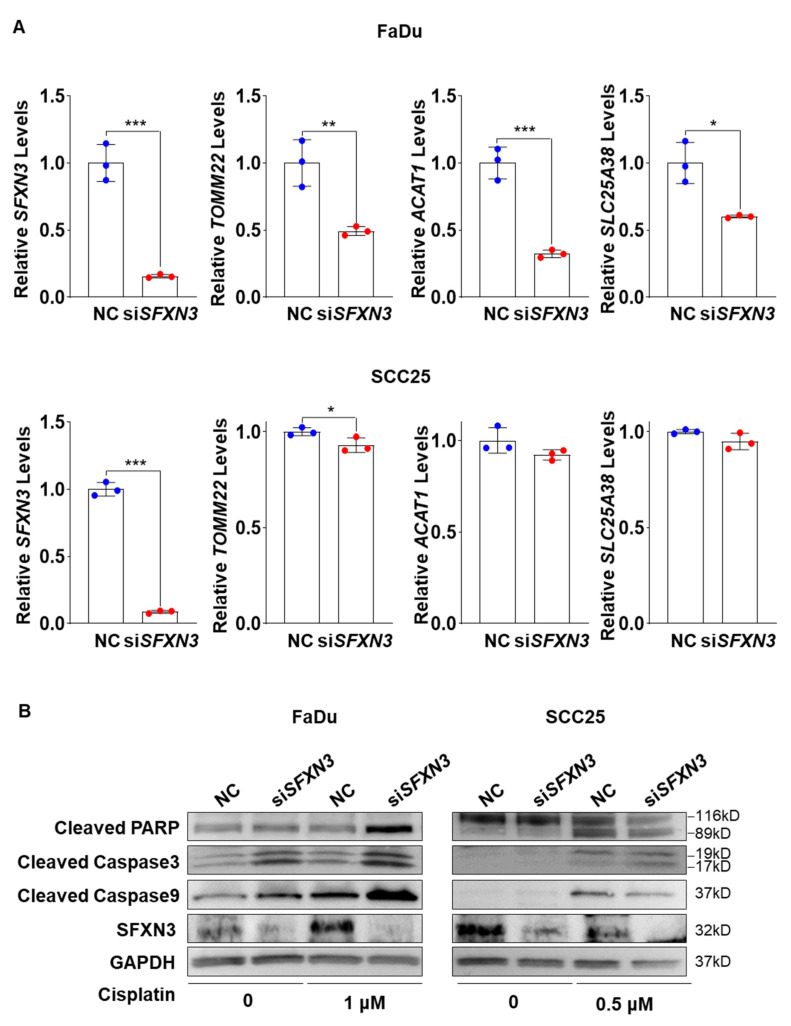
Association of SFXN3 depletion with altered mitochondrial-related gene expression and enhanced cisplatin-induced apoptotic signaling in HNSCC cells. (**A**) Relative mRNA expression levels of SFXN3, TOMM22, ACAT1, and SLC25A38 were measured in FaDu and SCC25 cells transfected with negative control siRNA (NC) or siSFXN3. SFXN3 knockdown was associated with a significant reduction in mitochondrial-related gene expression in FaDu cells, whereas SCC25 cells exhibited a more selective response, with TOMM22 showing the most consistent decrease. n = 3 (**B**) Western blot analysis of apoptosis-related proteins in FaDu and SCC25 cells transfected with NC or siSFXN3 and treated with cisplatin (FaDu, 1 μM; SCC25, 0.5 μM). Increased cleavage of PARP, caspase-3, and caspase-9 was observed following SFXN3 knockdown in both cell lines compared with NC-treated controls. GAPDH was used as a loading control. n = 3 Data are presented as mean ± standard deviation (SD). Statistical significance was determined using appropriate statistical tests. Statistical significance is indicated as *p < 0.05, **p < 0.01, and ***p < 0.001.

To explore the molecular pathways driving SFXN3-associated cisplatin responsiveness in HNSCC, a protein–protein interaction analysis (PPI) network analysis was conducted utilizing the STRING database. The resulting network identified SFXN3 as a central node interacting with several key mitochondrial-associated proteins, including TOMM22 (Translocase of Outer Mitochondrial Membrane 22), SLC2538A (Solute Carrier Family 25 member 38), and ACAT1 (Acetyl-CoA Acetyltransferase 1) (Fig. S4). Based on these predicted interactions, we next examined whether SFXN3 depletion affected the expression of these mitochondrial proteins. Quantitative expression analysis revealed that knockdown of SFXN3 significantly reduced the expression levels of TOMM22, ACAT1, and SLC25A38 in FaDu cells. In SCC25 cells, SFXN3 silencing resulted in a marked decrease in TOMM22 expression, whereas ACAT1 and SLC25A38 levels showed modest but consistent reductions (Fig. 9A). Given the involvement of these proteins in mitochondrial function, we further assessed apoptotic signaling following cisplatin treatment. Western blot analysis demonstrated that SFXN3 knockdown markedly enhanced cisplatin-induced activation of apoptosis, as evidenced by increased cleavage of PARP, caspase-3, and caspase-9 in both FaDu (1 μM cisplatin) and SCC25 (0.5 μM cisplatin) cells compared with negative control cells (Fig. 9B). Collectively, these results suggest that SFXN3 contributes to cisplatin resistance in HNSCC cells by regulating mitochondrial-associated protein expression and attenuating caspase-dependent apoptotic signaling, thereby linking SFXN3 expression to mitochondrial integrity and apoptotic responsiveness.

## 4 Discussion

This study provides a comprehensive evaluation of the clinical, molecular, immunological, and functional relevance of SFXN3 in HNSCC. By integrating large-scale transcriptomic data, immune infiltration profiles, and experimental validation, this study showed that SFXN3 is significantly overexpressed in HNSCC and strongly associated with poor clinical outcomes, pathological severity, altered metabolic status, and enhanced tumorigenic potential. Collectively, these findings indicate SFXN3 as an underrecognized oncogenic mediator and a promising biomarker with potential prognostic and therapeutic significance.

Across TCGA and multiple independent GEO cohorts, SFXN3 expression was consistently overexpressed in tumor tissues compared to normal tissues, indicating a robust transcriptional signature unaffected by dataset or platform differences. The consistent expression of SFXN3 indicates that it is an inherent feature of HNSCC. High SFXN3 levels were significantly associated with poorer overall and relapse-free survival across analyses and confirmed as an independent prognostic factor. The prognostic effect of SFXN3 was evident even in early-stage HNSCC, suggesting that it may drive oncogenesis from initiation and influence disease progression. Notably, our multivariate subgroup analysis indicated that the prognostic significance of SFXN3 was more pronounced in HPV-positive patients compared to HPV-negative patients, suggesting a potential interplay between SFXN3-mediated metabolic alteration and HPV infection status, although further investigation is needed (Supplementary Tables S4 and S5).

Beyond cancer, the SFXN family also regulates mitochondrial metabolism and cellular homeostasis [9,12,20]. SFXN proteins are conserved mitochondrial inner-membrane transporters that regulate amino acid metabolism, one-carbon metabolism, iron–sulfur cluster biogenesis, and mitochondrial iron homeostasis [8,9,20]. Dysregulation of these processes is linked to various pathological conditions, including congenital mitochondrial disorders, hematologic abnormalities, and neurodegenerative diseases [10,21,22,23]. Within this broader biological context, our findings indicate that aberrant SFXN3 expression in HNSCC may represent a tumor-specific exploitation of core mitochondrial metabolic pathways essential for cell survival and stress adaptation.

Consistent with these biological functions, emerging cancer studies report associations between SFXN family gene expression and patient prognosis across multiple cancers, including prostate cancer [12], breast cancer [24], hepatocellular carcinoma [25], lung adenocarcinoma [26], multiple myeloma [27], and acute myeloid leukemia [28]. High expression of individual SFXN members correlates with poorer overall and relapse-free survival in these malignancies. Analyses across pan-cancer and hematologic malignancies have linked increased SFXN3 expression to high-risk disease features and poor clinical outcomes, while recent transcriptomic and regulatory network studies report its prognostic relevance in HNSCC [29]. Collectively, these findings suggest that dysregulation of SFXN family-mediated mitochondrial metabolic programs may be a common feature of aggressive tumors, providing a broader oncologic context for the prognostic and functional significance of SFXN3 observed in this study.

A key finding of this study is the association between SFXN3 and immune infiltration patterns. High SFXN3 expression was associated with extensive remodeling of the tumor immune landscape, affecting the abundance and functional states of key immune cell populations. Since tumor-infiltrating immune cells strongly influence tumor progression, immune evasion, and therapy response, these findings indicate that SFXN3 may be associated with an immunosuppressive tumor microenvironment. However, it is crucial to note that high SFXN3 expression predicts poor survival in both immune-enriched and immune-depleted tumors. This independent prognostic value suggests that SFXN3’s intrinsic oncogenic functions—such as promoting cell proliferation and conferring cisplatin resistance via mitochondrial regulation—are the primary drivers of HNSCC progression. As immunotherapy becomes increasingly integrated into HNSCC management, identifying molecular determinants of immune behavior is clinical relevant. SFXN3 may serve as a biomarker to stratify patients based on immune phenotypes or identify those likely to benefit from metabolic- or immune-targeted combination therapies.

We also observed a strong positive correlation between SFXN3 expression and EGFR, a key oncogenic driver in HNSCC. Given the role of EGFR in proliferation, survival, angiogenesis, and therapeutic resistance, the coordinated upregulation of SFXN3 and EGFR suggests that SFXN3 may contribute to or enhance EGFR-driven oncogenic pathways. Since EGFR inhibitors are clinically used, the co-expression profile of SFXN3 and EGFR may provide additional prognostic or predictive value and support the potential for dual-targeted therapies.

Functional assays performed in this study confirmed the oncogenic role of SFXN3 in HNSCC. As shown in Fig. 7C and Supplementary Fig. S3, siRNA-mediated knockdown of SFXN3 significantly inhibited cell proliferation and clonogenicity. Furthermore, SFXN3 depletion sensitized cells to cisplatin, as evidenced by markedly elevated cleavage levels of PARP, caspase-3, and caspase-9 (Fig. 9B), indicating the activation of the intrinsic apoptotic pathway. These mechanistic observations are consistent with the biological functions of SFXN3 as a mitochondrial transporter involved in metabolic homeostasis. Since cancer cells depend on mitochondrial reprogramming to sustain growth and evade apoptosis, our findings suggest that SFXN3 acts as a key regulator shaping tumor cell survival and chemoresponsiveness.

Notably, despite the consistent phenotypic effects observed across cell lines, the molecular consequences of SFXN3 depletion differed between FaDu and SCC25 cells, highlighting cell line–specific heterogeneity in HNSCC. FaDu cells exhibited a pronounced downregulation of multiple mitochondrial-associated genes following SFXN3 knockdown, whereas SCC25 cells showed a more selective response, with TOMM22 being the most consistently affected target [30].

These differences may reflect intrinsic biological distinctions between the two cell lines, including variations in cellular origin, metabolic dependency, mitochondrial activity, and baseline apoptotic threshold. FaDu cells, derived from hypopharyngeal carcinoma, are reported to display higher proliferative capacity and metabolic activity, whereas SCC25 cells, originating from tongue squamous cell carcinoma, often exhibit more differentiated epithelial characteristics. Such intrinsic differences may influence the extent to which SFXN3 depletion perturbs mitochondrial-associated gene expression and downstream apoptotic signaling.

Importantly, despite these cell line–specific differences at the gene expression level, SFXN3 knockdown consistently enhanced cisplatin-induced apoptosis in both FaDu and SCC25 cells. This convergence at the phenotypic level suggests that SFXN3 may contribute to cisplatin responsiveness through partially overlapping but context-dependent mechanisms across distinct HNSCC cellular backgrounds [31].

Overall, our findings position SFXN3 as a central molecular determinant in HNSCC, influencing tumor initiation, metabolism, immune landscape remodeling, proliferation, and treatment response. Although further mechanistic studies—particularly those incorporating *in vivo* models—are warranted, the present data establish *SFXN3* as both a valuable prognostic indicator and a viable therapeutic vulnerability in high-risk HNSCC.

### Limitations

Despite the strengths of combining multi-platform datasets with functional validation, some limitations remain. First, the retrospective design of the transcriptomic analyses may introduce biases from sample heterogeneity, sequencing depth, and variability in clinical annotations. Consistent SFXN3 upregulation across multiple cohorts supports these findings, but prospective validation in well-controlled clinical samples is still required. Second, while correlations between SFXN3, immune infiltration, and EGFR expression were strong, the underlying mechanistic pathways remain unclear. Whether SFXN3 directly regulates these processes or reflects downstream oncogenic signaling remains unclear. Third, functional validation was mainly conducted in FaDu cells, which may not capture the molecular diversity of all HNSCC subtypes. Including additional cell lines or patient-derived organoids would provide a more comprehensive understanding of SFXN3-mediated effects. Finally, the association between body weight and SFXN3 expression warrants further investigation of metabolic and inflammatory signaling, though these associations remain correlative without mechanistic assays addressing obesity-related pathways.

## 5 Conclusion

This study shows SFXN3 as a clinically significant and functionally active oncogene in HNSCC. SFXN3 is consistently upregulated across multiple patient cohorts and is associated with poor prognosis, advanced pathological states, increased EGFR expression, and clinical metabolic parameters, including higher body weight. Its association with immune infiltration indicates a role in creating an immunosuppressive tumor microenvironment, while *in vitro* experiments reveal that SFXN3 promotes proliferation, chemoresistance, and long-term clonogenic survival. Together, these findings indicate SFXN3 as a promising biomarker for risk stratification and a potential therapeutic target. Therefore, future studies should clarify the mechanistic pathways regulated by SFXN3 and assess its potential for targeted or combination therapies to improve outcomes for patients with HNSCC.

## Data Availability

All datasets used in this study are publicly available in TCGA, GEO, and other referenced platforms.

## Supplementary Materials

The supplementary material is available online at https://www.techscience.com/doi/10.32604/or.2026.078376/s1.

## Nomenclature

SFXN3	Sideroflexin 3
HNSCC	head and neck squamous cell carcinoma
TCGA	The Cancer Genome Atlas
GEO	Gene Expression Omnibus
SFXN	sideroflexin
OS	overall survival
HR	hazard ratio
CI	confidence interval
CPTAC	Clinical Proteomic Tumor Analysis Consortium
siRNA	small interfering RNA
NC	negative control
qRT-PCR	quantitative Real-Time Polymerase Chain Reaction
SARC	Sarcoma
PAAD	Pancreatic adenocarcinoma
KIRP	Kidney renal papillary cell carcinoma
KIRC	Kidney renal clear cell carcinoma
EGFR	epidermal growth factor receptor
PI	propidium iodide
NKT	natural killer T-cell
T-reg	Regulatory T-cell
