# Supplementary material for: SFXN3 Serves as a Predictive Biomarker for Cisplatin Response and Survival in Head and Neck Squamous Cell Carcinoma
Source: Oncol Res. 2026 May 21;34(6):22. doi: 10.32604/or.2026.078376 (PMC13223184; doi:10.32604/or.2026.078376)
Supplement: Supplementary file 1 [file OncolRes-34-78376-s001.zip › TSP_OR_78376-s001.docx]

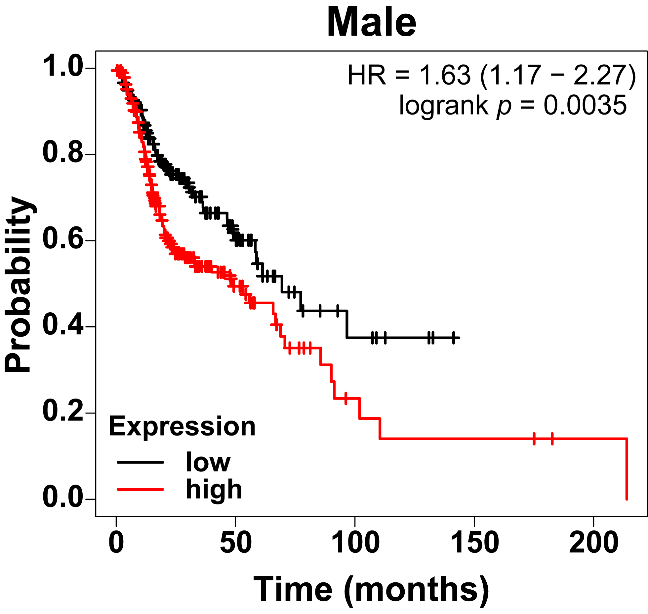

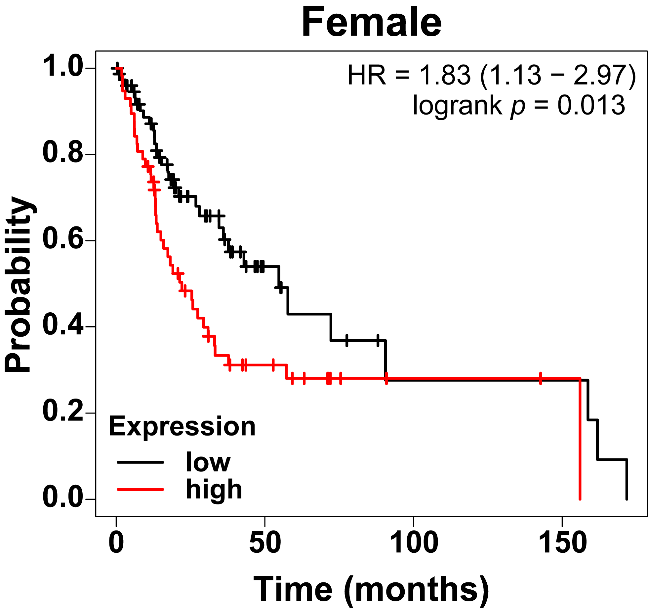

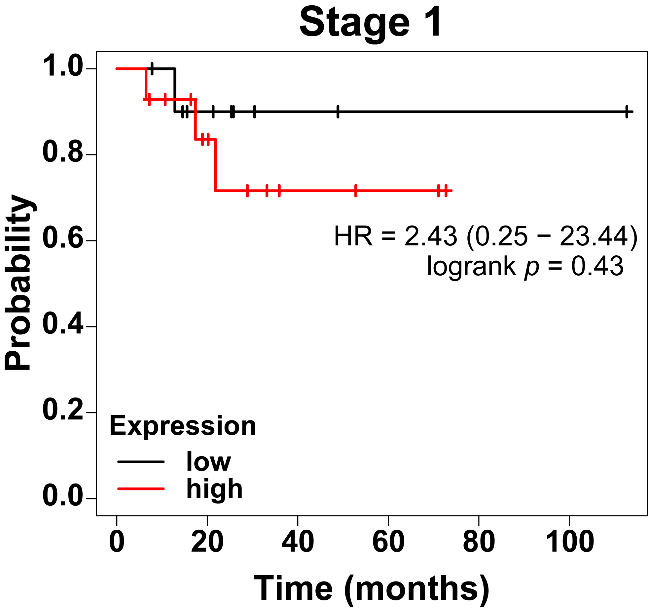

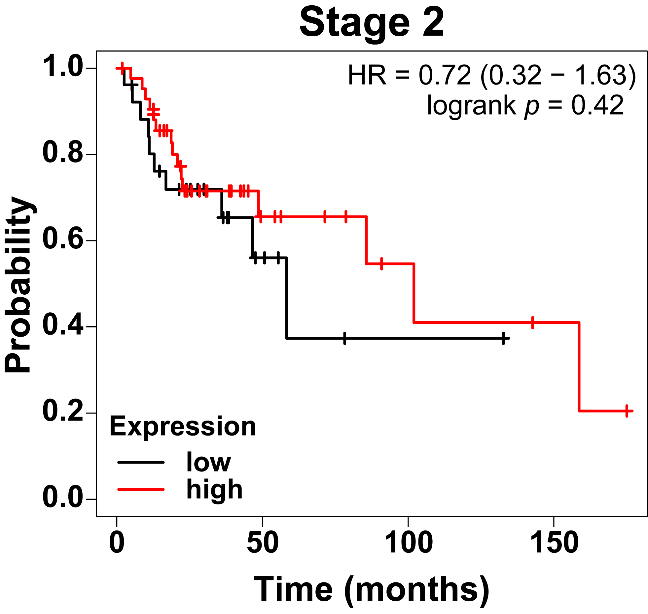

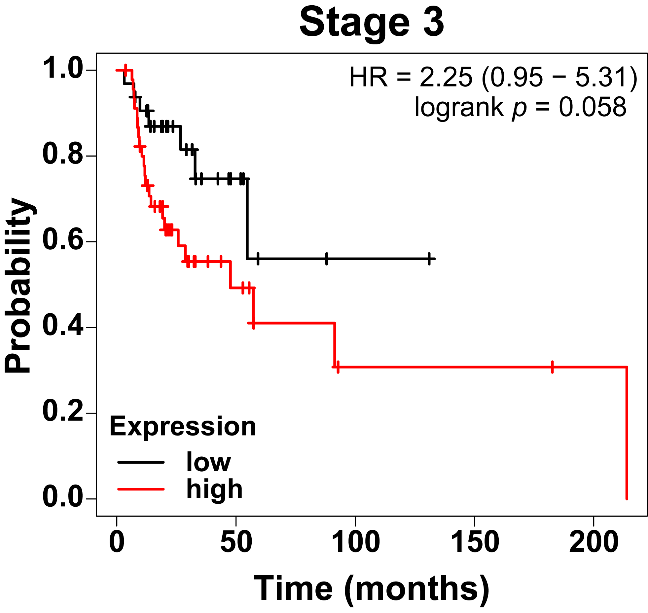

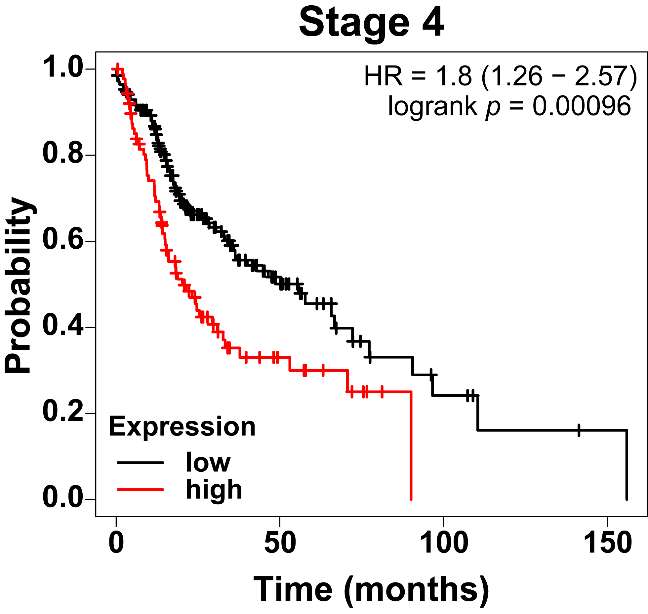


**C**

**D**

**E**

**F**

**A**

**B**

**Supplementary Figure S1.** Prognostic significance of SFXN3 expression stratified by sex and clinical stage in HNSCC. (A) Male patients: elevated SFXN3 expression is significantly associated with decreased OS (HR = 1.63, 95% CI: 1.17–2.27, log-rank p = 0.0035). (B) Female patients: high SFXN3 expression similarly predicts significantly worse OS (HR = 1.83, 95% CI: 1.13–2.97, log-rank p = 0.013). (C) Stage 1 tumors: high SFXN3 expression shows an adverse, but statistically nonsignificant, trend toward poorer OS (HR = 2.43, 95% CI: 0.25–23.44, log-rank p = 0.43). (D) Stage 2 tumors: high SFXN3 expression shows a nonsignificant favorable trend toward improved OS (HR = 0.72, 95% CI: 0.32–1.63, log-rank p = 0.42). (E) Stage 3 tumors: high SFXN3 expression shows a strong trend toward poorer OS (HR = 2.25, 95% CI: 0.95–5.31, log-rank p = 0.058). (F) Stage 4 tumors: Elevated SFXN3 levels strongly predicted a substantial decline in overall survival (HR = 1.8, 95% CI: 1.26–2.57, log-rank p = 0.00096).


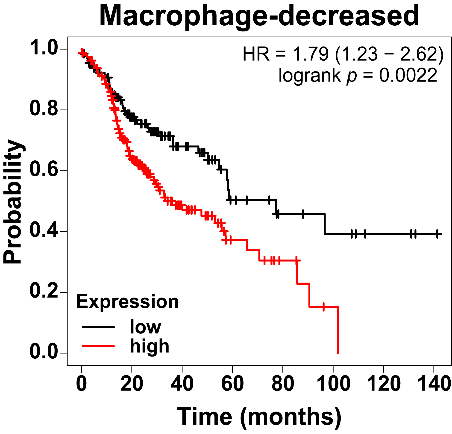

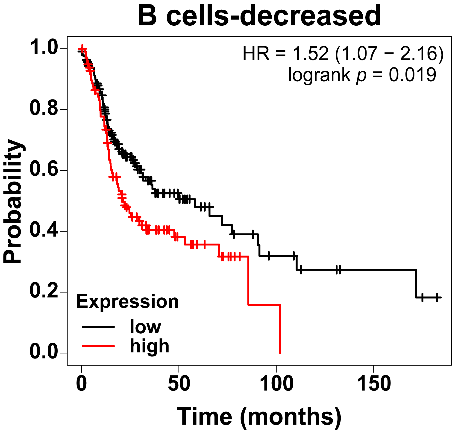

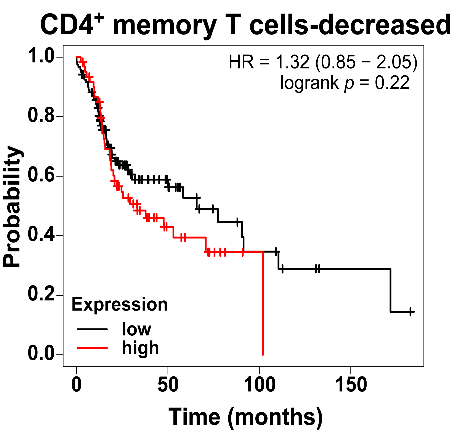

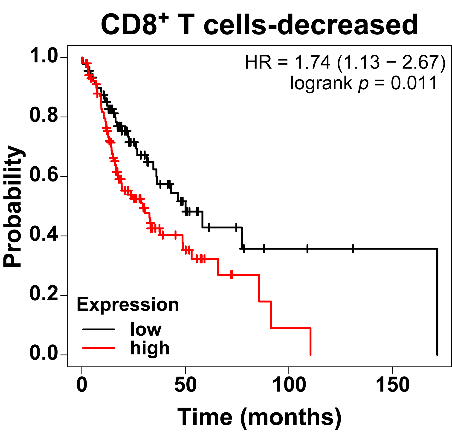


**C**

**D**

**A**

**B**


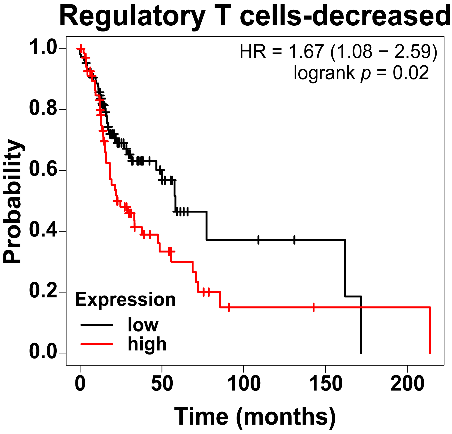

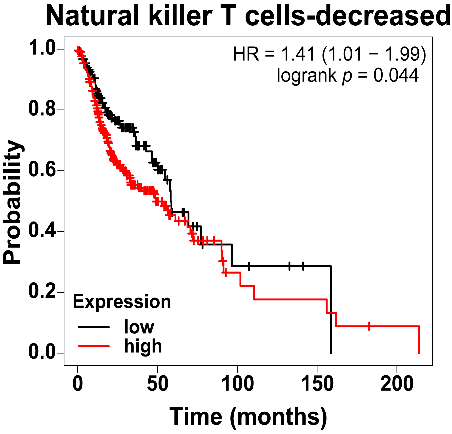

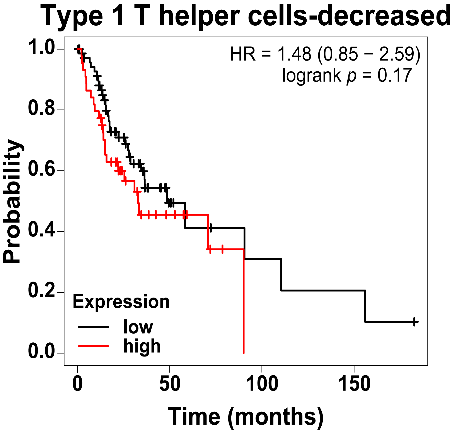

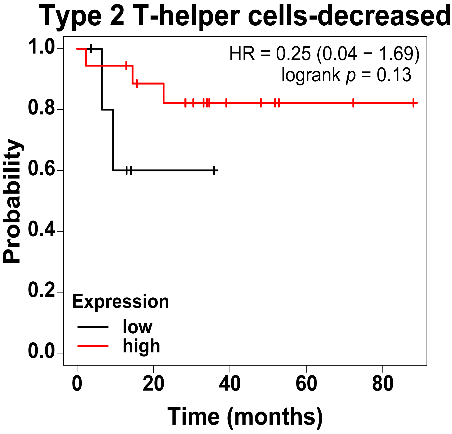


**G**

**H**

**E**

**F**

**Supplementary Figure S2.** Survival analysis of SFXN3 expression in tumors with decreased immune cell infiltration. (A) Kaplan–Meier analysis revealing that in macrophage-depleted tumors, high SFXN3 expression is associated with reduced OS (HR = 1.79, log-rank p = 0.0022). (B) B-cell–depleted tumors: elevated SFXN3 predicts poorer OS (HR = 1.52, p = 0.019). (C) CD4⁺ memory T-cell–depleted tumors: high SFXN3 shows a nonsignificant trend toward worse OS (HR = 1.32, p = 0.22). (D) CD8⁺ T-cell–depleted tumors: high SFXN3 expression is associated with significantly reduced OS (HR = 1.74, p = 0.011). (E) T-reg–depleted tumors: high SFXN3 corresponds to poorer OS (HR = 1.67, p = 0.02). (F) NKT-cell–depleted tumors: high SFXN3 expression is linked to inferior OS (HR = 1.41, p = 0.044). (G) Type I T-helper cell–depleted tumors: high SFXN3 shows no significant adverse trend (HR = 1.48, p = 0.17). (H) Type II T-helper cell–depleted tumors: high SFXN3 shows no significant survival association (HR = 0.25, p = 0.13).


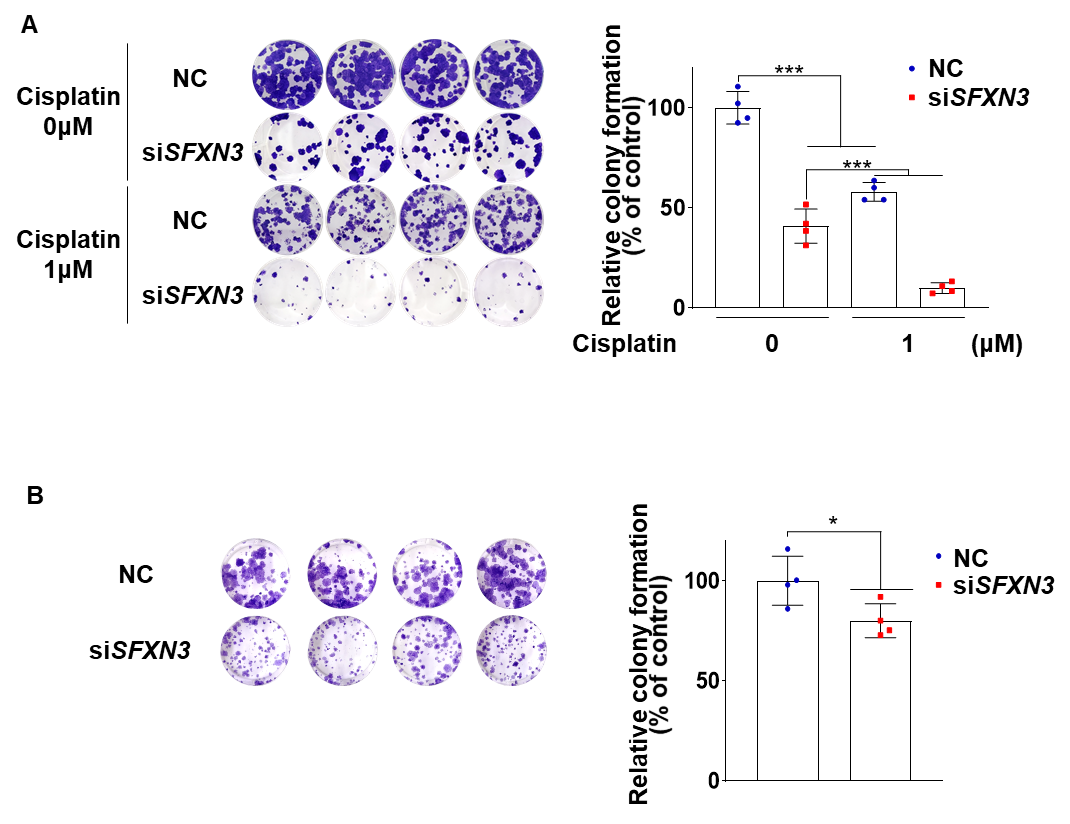


**Supplementary Figure S3.** Effect of SFXN3 knockdown on clonogenic survival in HNSCC cells. (A) Representative images (left) and quantitative analysis (right) of colony formation assays in FaDu cells transfected with negative control siRNA (NC) or siSFXN3 and treated with cisplatin (0 or 1 μM). The depletion of SFXN3 markedly impaired clonogenic survival, a suppressive effect that was further potentiated upon exposure to cisplatin. (B) Colony formation assay in SCC25 cells transfected with NC or siSFXN3 in the absence of cisplatin. SFXN3 depletion alone resulted in a moderate but significant decrease in clonogenic survival compared with control cells. Data are presented as mean ± standard deviation (SD). Statistical significance was determined using appropriate statistical tests. Statistical significance is indicated as *p < 0.05 and ***p < 0.001.


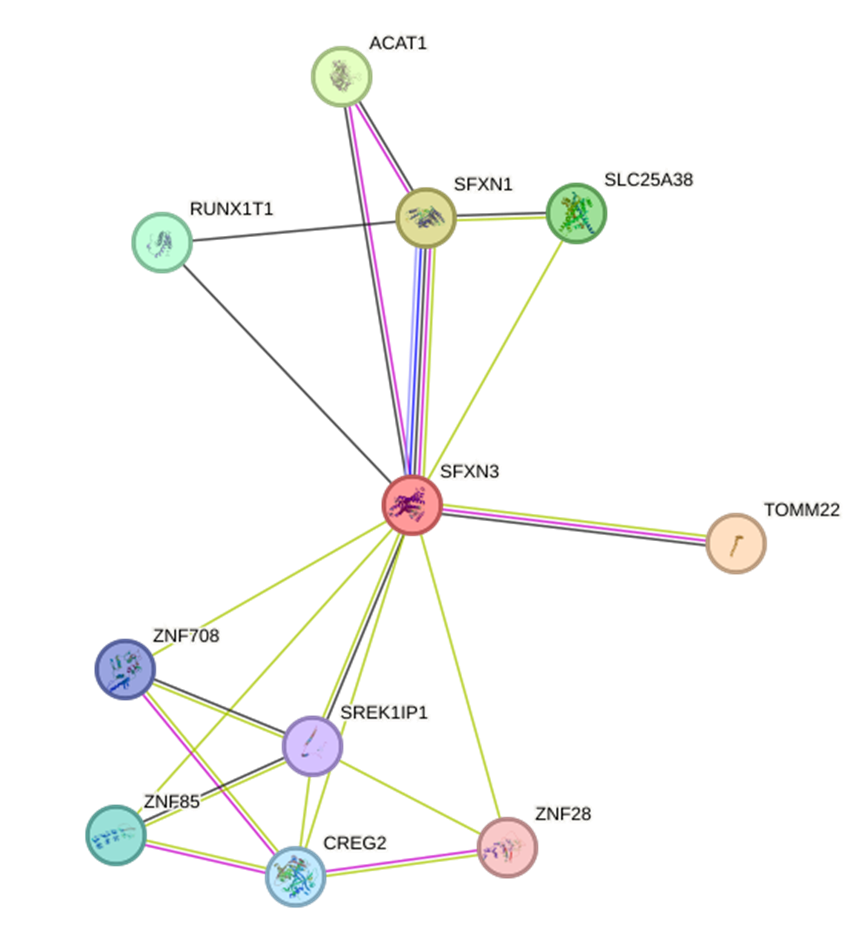


**Supplementary Figure S4.** STRING-based protein–protein interaction network of SFXN3.

To identify potential molecular binding partners and functional pathways associated with SFXN3, a protein-protein interaction (PPI) network was generated utilizing the STRING platform. The network illustrates predicted interactions between SFXN3 and proteins involved in mitochondrial function and metabolic processes, including TOMM22, ACAT1, and SLC25A38, as well as additional proteins implicated in cellular regulation. Edges represent known or predicted protein–protein associations derived from curated databases, experimental evidence, and computational predictions. This network analysis was used as a hypothesis-generating approach to identify potential pathways associated with SFXN3 in HNSC.

**Supplementary Table S1. Multivariate Cox regression analysis of SFXN3 and clinical characteristics with overall survival in HNSCC patients (n=522).**

| Variable | Coefficient | Hazard Ratio (HR) | 95% CI | p-value |
| --- | --- | --- | --- | --- |
| **SFXN3 (Expression)** | **0.214** | **1.239** | **1.023 – 1.500** | **0.028*** |
| **Age** | **0.023** | **1.024** | **1.009 – 1.039** | **0.002**** |
| Gender |  |  |  |  |
| Female |  | 1.000 |  |  |
| Male | -0.253 | 0.777 | 0.554 – 1.089 | 0.143 |
| Race |  |  |  |  |
| Others |  | 1.000 |  |  |
| Black | 0.129 | 1.138 | 0.381 – 3.403 | 0.817 |
| White | -0.212 | 0.809 | 0.297 – 2.203 | 0.679 |
| Stage |  |  |  |  |
| Stage 1 |  | 1.000 |  |  |
| Stage 2 | 0.615 | 1.849 | 0.637 – 5.367 | 0.258 |
| Stage 3 | 0.887 | 2.429 | 0.848 – 6.958 | 0.098 |
| **Stage 4** | **1.273** | **3.570** | **1.313 – 9.704** | **0.013*** |
| Tumor Purity | 0.139 | 1.149 | 0.554 – 2.380 | 0.709 |

*CI: Confidence Interval. * p < 0.05, ** p < 0.01.

**Supplementary Table S2. Statistical analysis of apoptosis in FaDu cells.**

Two-way ANOVA and Sidak’s multiple comparisons (NC vs siSFXN3 × non-treated vs Cisplatin)

| Endpoint | Comparison | Mean difference | 95% CI | Adjusted P | Significant |
| --- | --- | --- | --- | --- | --- |
| Live cell  (%) | NC: non vs Cis | 12.69 | 11.82→13.57 | <0.0001 | **** |
|  | siSFXN3: non vs Cis | 20.28 | 19.41→21.15 | <0.0001 | **** |
|  | Non: NC vs siSFXN3 | 11.05 | 10.17→11.92 | <0.0001 | **** |
|  | Cis: NC vs siSFXN3 | 18.63 | 17.76→19.51 | <0.0001 | **** |
| Early apoptosis (%) | NC: non vs Cis | -4.003 | -4.878 → -3.129 | <0.0001 | **** |
|  | siSFXN3: non vs Cis | -5.707 | -6.581 → -4.832 | <0.0001 | **** |
|  | Non: NC vs siSFXN3 | −5.683 | −6.558 → −4.809 | <0.0001 | **** |
|  | Cis: NC vs siSFXN3 | −7.387 | −8.281 → −6.512 | <0.0001 | **** |
| Late apoptosis (%) | NC: non vs Cis | −8.220 | −9.095 → −7.345 | <0.0001 | **** |
|  | siSFXN3: non vs Cis | −14.45 | −15.32 → −13.58 | <0.0001 | **** |
|  | Non: NC vs siSFXN3 | -4.580 | −5.435 → −3.685 | <0.0001 | **** |
|  | Cis: NC vs siSFXN3 | −10.79 | −11.68 to −9.915 | <0.0001 | **** |
| Necrosis  (%) | NC: non vs Cis | −0.593 | −1.488 → 0.281 | 0.3372 | ns |
|  | siSFXN3: non vs Cis | -0.973 | -1.848 → -0.099 | 0.0227 | * |
|  | Non: NC vs siSFXN3 | −0.213 | −1.088 → 0.861 | 0.9842 | ns |
|  | Cis: NC vs siSFXN3 | −0.593 | −1.488 → 0.281 | 0.3372 | ns |

* p<0.05, ****p<0.0001, ns no significance.

**Supplementary Table S3. Statistical analysis of apoptosis in SCC25 cells.**

Two-way ANOVA and Sidak’s multiple comparisons (NC vs siSFXN3 × non-treated vs Cisplatin)

| Endpoint | Comparison | Mean difference | 95% CI | Adjusted P | Significant |
| --- | --- | --- | --- | --- | --- |
| Live cell (%) | NC: non vs Cis | 21.77 | 16.42 → 27.12 | <0.0001 | **** |
|  | siSFXN3: non vs Cis | 33.01 | 27.66 → 38.36 | <0.0001 | **** |
|  | Non: NC vs siSFXN3 | 4.61 | -0.7416 → 9.962 | 0.1577 | ns |
|  | Cis: NC vs siSFXN3 | 15.84 | 10.49 → 21.19 | 0.0003 | *** |
| Early apoptosis (%) | NC: non vs Cis | -9.28 | -11.83 → -6.731 | <0.0001 | **** |
|  | siSFXN3: non vs Cis | -21.16 | -23.71 → -18.61 | <0.0001 | **** |
|  | Non: NC vs siSFXN3 | -2.77 | -5.319 → -0.221 | 0.0719 | ns |
|  | Cis: NC vs siSFXN3 | -14.65 | -17.2 → -12.1 | <0.0001 | **** |
| Late apoptosis (%) | NC: non vs Cis | -11.05 | -13.9 → -8.204 | <0.0001 | **** |
|  | siSFXN3: non vs Cis | -14.56 | -17.41 → -11.71 | <0.0001 | **** |
|  | Non: NC vs siSFXN3 | -1.6333 | -4.482 → 1.216 | 0.3958 | ns |
|  | Cis: NC vs siSFXN3 | -5.1433 | -7.992 → -2.294 | 0.0063 | ** |
| Necrosis (%) | NC: non vs Cis | -0.3833 | -0.5339 → -0.2328 | 0.0007 | *** |
|  | siSFXN3: non vs Cis | -0.1833 | -0.3339 → -0.0328 | 0.0453 | * |
|  | Non: NC vs siSFXN3 | -0.16 | -0.3105 → -0.009471 | 0.0781 | ns |
|  | Cis: NC vs siSFXN3 | 0.04 | -0.1105 → 0.1905 | 0.8038 | ns |

* p<0.05, ** p<0.01, ***p<0.001, ****p<0.0001, ns no significance.

**Supplementary Table S4. Multivariate Cox regression analysis in HPV-positive HNSCC patients (n=98).**

| Variable | Coefficient | Hazard Ratio (HR) | 95% CI | p-value |
| --- | --- | --- | --- | --- |
| **SFXN3 (Expression)** | **0.833** | **2.301** | **1.322 – 4.003** | **0.003**** |
| Age | 0.022 | 1.023 | 0.974 – 1.073 | 0.365 |
| Gender |  |  |  |  |
| Female |  | 1.000 |  |  |
| Male | -0.426 | 0.653 | 0.232 – 1.841 | 0.420 |
| Tumor Purity | -1.719 | 0.179 | 0.021 – 1.537 | 0.117 |
| Stage/Race | N/A | N/A | N/A | > 0.999† |

*CI: Confidence Interval. ** p < 0.01. †Due to the limited sample size and number of events in the HPV-positive cohort, the hazard ratios for Stage and Race could not be reliably estimated (statistical convergence failure).

**Supplementary Table S5. Multivariate Cox regression analysis in HPV-negative HNSCC patients (n=422).**

| Variable | Coefficient | Hazard Ratio (HR) | 95% CI | p-value |
| --- | --- | --- | --- | --- |
| SFXN3 (Expression) | 0.144 | 1.155 | 0.929 – 1.435 | 0.194 |
| **Age** | **0.028** | **1.029** | **1.012 – 1.046** | **0.001**** |
| Gender |  |  |  |  |
| Female |  | 1.000 |  |  |
| Male | -0.279 | 0.757 | 0.529 – 1.083 | 0.128 |
| Race |  |  |  |  |
| Others |  | 1.000 |  |  |
| Black | -0.011 | 0.989 | 0.327 – 2.989 | 0.985 |
| White | -0.378 | 0.685 | 0.251 – 1.871 | 0.461 |
| Stage |  |  |  |  |
| Stage 1 |  | 1.000 |  |  |
| Stage 2 | 0.363 | 1.437 | 0.486 – 4.256 | 0.512 |
| Stage 3 | 0.758 | 2.134 | 0.738 – 6.167 | 0.162 |
| **Stage 4** | **1.16** | **3.190** | **1.169 – 8.704** | **0.024*** |
| Tumor Purity | 0.328 | 1.388 | 0.623 – 3.091 | 0.422 |

*CI: Confidence Interval. * p < 0.05, ** p < 0.01.
